# A Custom EOG-Based HMI Using Neural Network Modeling to Real-Time for the Trajectory Tracking of a Manipulator Robot

**DOI:** 10.3389/fnbot.2020.578834

**Published:** 2020-09-29

**Authors:** Francisco D. Perez Reynoso, Paola A. Niño Suarez, Oscar F. Aviles Sanchez, María B. Calva Yañez, Eduardo Vega Alvarado, Edgar A. Portilla Flores

**Affiliations:** ^1^Instituto Politécnico Nacional, Escuela Superior de Ingeniería Mecánica y Eléctrica, Mexico City, Mexico; ^2^Departamento de Ingeniería Mecatrónica, Universidad Militar Nueva Granada, Bogotá, Colombia; ^3^Centro de Innovación Tecnológica en Computo, Instituto Politécnico Nacional, Mexico City, Mexico

**Keywords:** EOG, HMI, customization calibration, MNN, optimization, robots trajectories

## Abstract

Although different physiological signals, such as electrooculography (EOG) have been widely used in the control of assistance systems for people with disabilities, customizing the signal classification system remains a challenge. In most interfaces, the user must adapt to the classification parameters, although ideally the systems must adapt to the user parameters. Therefore, in this work the use of a multilayer neural network (MNN) to model the EOG signal as a mathematical function is presented, which is optimized using genetic algorithms, in order to obtain the maximum and minimum amplitude threshold of the EOG signal of each person to calibrate the designed interface. The problem of the variation of the voltage threshold of the physiological signals is addressed by means of an intelligent calibration performed every 3 min; if an assistance system is not calibrated, it loses functionality. Artificial intelligence techniques, such as machine learning and fuzzy logic are used for classification of the EOG signal, but they need calibration parameters that are obtained through databases generated through prior user training, depending on the effectiveness of the algorithm, the learning curve, and the response time of the system. In this work, by optimizing the parameters of the EOG signal, the classification is customized and the domain time of the system is reduced without the need for a database and the training time of the user is minimized, significantly reducing the time of the learning curve. The results are implemented in an HMI for the generation of points in a Cartesian space (*X, Y, Z*) in order to control a manipulator robot that follows a desired trajectory by means of the movement of the user's eyeball.

## Introduction

The development of human–machine Interfaces (HMI) has been on the rise due to the incorporation of physiological signals as inputs to the control algorithms. Currently, robots are collaborative and interact with humans to improve their quality of life, which has allowed the development of intuitive interfaces for human–robot collaboration, in tasks, such as assistance and robotic rehabilitation. One of the study objectives in these systems is shared control, where a robotic system and human control the same body, tool, mechanism, etc. Shared control has originated in research fields, such as human–robot co-adaptation, where the two agents can benefit by each other's skills or must adapt to the other's behavior, to achieve the execution of effective cooperative tasks.

In this paper, it was considered that the human and individual characteristics affect the execution of the task that the HMI perform; these parameters are highly variable, and it is required to analyze and reduce the effects on the efficiency of the system. It is difficult to determine the level of adaptability or personalization of an HMI; however, calibrating a system looking for it to adapt to the personal parameters of a user has been shown to decrease the learning curve, improving the level of acceptance of inexperienced users. The proposed HMI will be implemented in the future to assist people with severe disabilities, where a manipulator robot will be adapted to a wheelchair, so that the user can control the movements of the robot by means of orientation of the gaze with the ability of taking objects and increasing their autonomy.

The work presented proposes to develop an intelligent calibration system to personalize the use of an HMI, where using EOG signals controls the trajectory tracking of a manipulator robot in its workspace. To achieve this, a fuzzy inference system is calibrated using the EOG signal of each user. The individual EOG signal was modeled by means of an MNN, implementing descending backpropagation using the Widrow–Hoff technique, obtaining a mathematical function that describes the waveform of the signal discrete EOG. The objective function obtained by means of the neural network is optimized using genetic algorithms to obtain the maximum and minimum voltage threshold of the EOG signal corresponding to each person. Once the variability range is obtained by optimizing the EOG signal, the fuzzy classifier is calibrated for the generation of coordinates in the Cartesian space (*X, Y, Z*). Gaussian membership functions define position in space by detecting EOG signal voltage thresholds; each threshold corresponds to a point in space defined precisely by calibration for each individual. In this case, a database is not required for the system to work; in most interfaces, they have a set of signals stored, and through training the user it is expected to reach the expected values, which only then does the system respond.

In section Overview of Related Work of this document, a summary of related works is presented; section Materials and Methods provides an overview of the neural network for non-linear regression of discrete EOG signal samples and details of the method used to implement the calibration system using genetic algorithms. The experimental procedure and analysis of results are presented in section Experiments and Results Analysis, and section Conclusion concludes the current work and discusses the advantages and limitations of the proposed system.

## Overview of Related Work

People with severe disabilities cannot move their lower and upper extremities, so designing interfaces with custom features has become a technological challenge (Lum et al., 2012); for this reason, controllers have been implemented that can adapt to the needs of the user using haptic algorithms, multimodal human–machine interfaces (mHMI) and incorporation of artificial intelligence algorithms (Dipietro et al., 2005) among others. In Gopinathan et al. (2017), a study is presented that describes the physical human–robot interaction (pHRI) using a custom rigidity control system of a 7-DOF KUKA industrial robot; the system is calibrated using a force profile obtained through each user and validates their performance by 49 participants using a heuristic control. A similar control system is applied in Buchli et al. (2011), where the level of force of each user is adapted to the control of a 3-DOF robot by haptics and is adjusted to the biomechanics of the user, in order to work on cooperative environments with humans (Gopinathan et al., 2017).

To customize assistive systems, Brain–Computer Interface (BCI) systems have also been developed in combination with electroencephalography (EEG), electromyography (EMG), and electrooculography (EOG) signals (Ang et al., 2015). In Zhang et al. (2019), a multimodal system (mHMI) is presented that can achieve a classification accuracy of physiological signals with an average of 93.83%, which is equivalent to a control speed of 17 actions per minute; the disadvantage that it presents is the long training time and the excessive use of sensors placed on the user. In Rozo et al. (2015), Gaussian functions are used to classify and learn cooperative human–robot skills in the context of object transport. In Medina et al. (2011), a method is proposed using Markov models to increase the experience of a manipulator robot in collaborative tasks with humans; the control adapts and improves cooperation through user speech commands and repetitive haptic training tasks.

The disadvantages of handling EEG for the development of Brain–Computer Interfaces (BCI) are described in Xiao and Ding (2013), since EEG signals do not have sufficient resolution because they attenuate during transmission; however, detection is reported in the EEG bandwidth using artificial intelligence that decodes individual finger movements for the control of prostheses. Advanced methods have been used for the detection, processing, and classification of EMG signals generated by muscle movements. In Gray et al. (2012), a comprehensive review was conducted on the changes that occur in the muscle after clinical alterations and how it affects the characteristics of the EMG signal, emphasizing the adaptability of the signal classification due to muscle injuries.

In the case of wheelchair control in Djeha et al. (2017), they use wavelet transform and an MNN for the classification of EOG and EEG signals, obtaining a classification accuracy rate of 93%; the classifier works in a control system for a virtual wheelchair. In Kumar et al. (2018), a review of human–computer interface systems based on EOG is presented; the work of 41 authors is explained, where the interfaces used implement artificial intelligence algorithms for signal classification. To calibrate the classifier, they use databases that contain an average of the signal threshold; they are characterized by implementing pattern search algorithms so that the machines designed to provide assistance have a response.

The HMI system presented in this paper has the property of being calibrated in real time, so it can be adapted to an EOG signal of any user, without the need for a database, unlike most of the systems reported in Kumar et al. (2018). The HMI works with any inexperienced user because of its capability of adapting to personal characteristics after a brief training. This is mainly due to the use of a calibration system designed from a multilayer neural network (MNN) to model the EOG signal as a mathematical function. The proposal in this work is that the user is not the one that adapts to previously acquired signals to generate a response in the system and that the system is the one that adapts to personal parameters of any user with severe motor disability. The preliminary results, obtained with 60 different users without disabilities in order to measure the adaptability of the system, showed that it was possible to generate trajectories to control a robot by means of ocular commands.

## Materials and Methods

**Figure 1:**
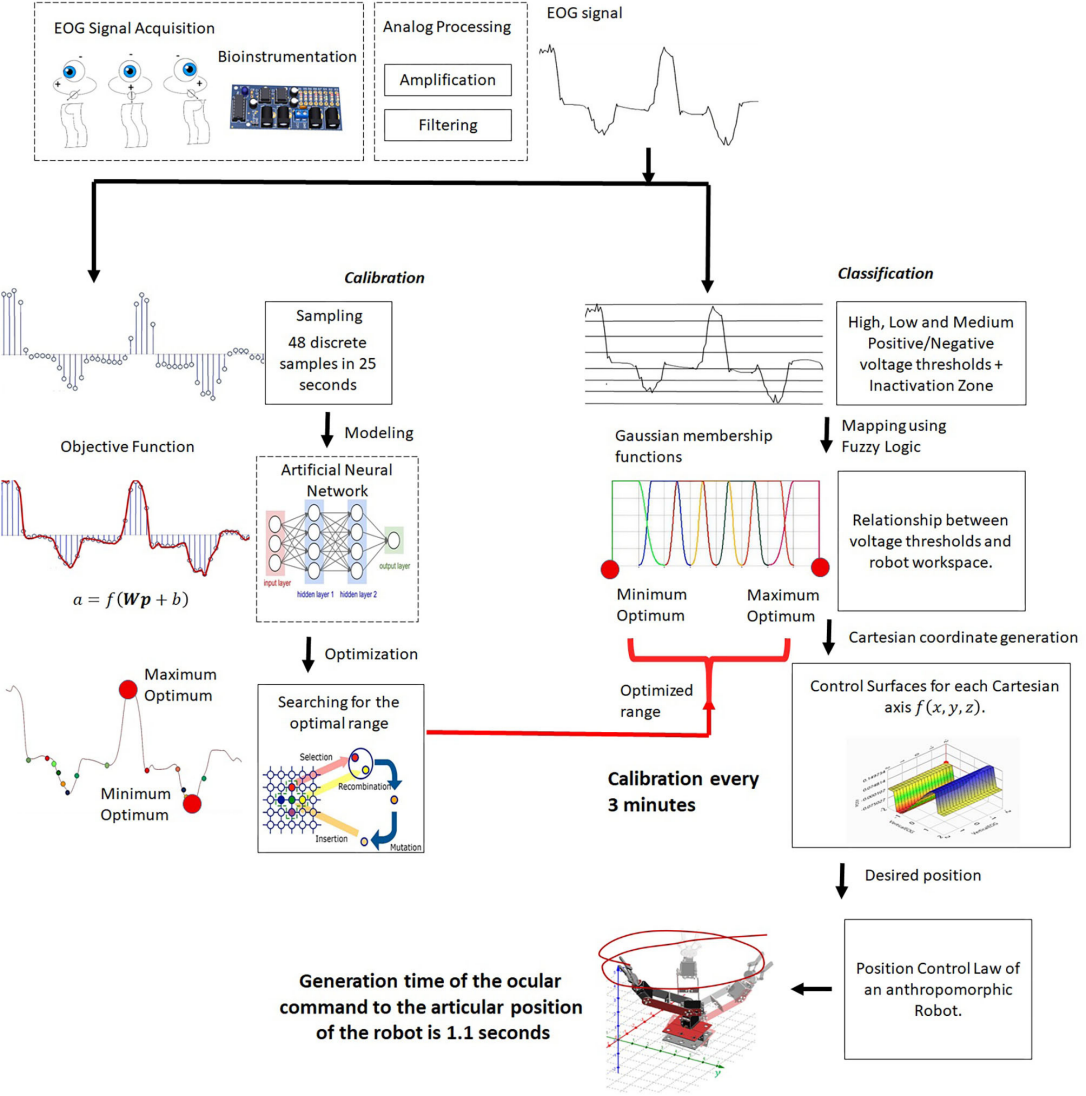
HMI system.

The developed system is presented in Figure 1; there is an analog acquisition stage of the EOG signal and two parallel processing of the signal. One is for classification, where the EOG signal is divided by means of voltage thresholds, and a fuzzy inference system is implemented to establish the relationship between the EOG signal and the workspace of an assistance robot. The classification using fuzzy logic requires a working threshold to generate points in the Cartesian space; these data represent the desired position to which a manipulating robot must arrive.

The other is for calibration of the fuzzy inference system. The proposed method is to obtain a mathematical model that describes the behavior of the EOG signal for each individual, and an algorithm that detects the optimal thresholds with which the classifier can be modified and adapted to any user. Customizing the control of the assistance system reduces time training necessary for mastering it. Thus, the objective function for each individual is obtained by optimizing the range of signal variability. These data are the input to the fuzzy classifier, adapting the interface to the personal properties of each user.

The proposed methodology for the development of the HMI consists of parallel processing, while acquisition digital processing and classification by means of fuzzy inference is carried out with a time from the generation of the eye movement to the articular movement of the robot of 1.1 s. Calibration consisting of modeling and optimization of the EOG signal is carried out every 3 min. When the optimal range data is obtained, it is transmitted through a virtual port, communicating the optimization results with the fuzzy classifier. So the range of the classifier is constantly updating, adapting to changes in either the signal, due to user changes, or in the variability of the voltage threshold due to fatigue and clinical alterations.

### EOG Acquisition

**Figure 2:**
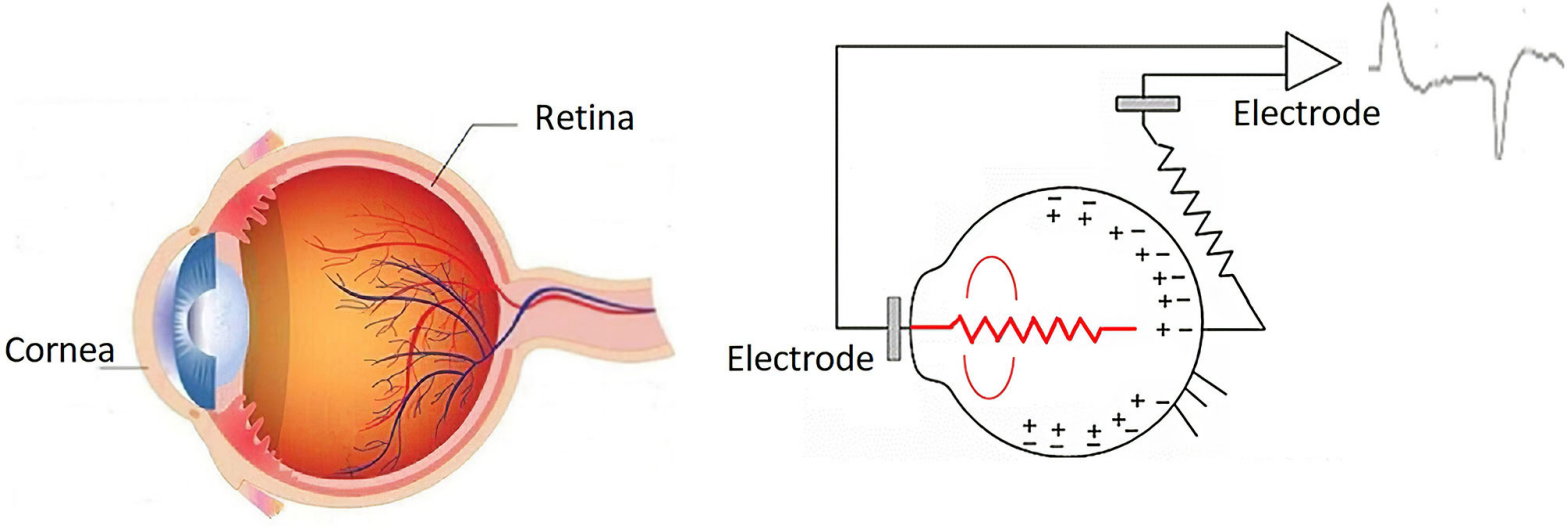
Retina–cornea action potential (Ding and Lv, 2020).

By generating an eye movement through the direct central position toward the periphery, the retina approaches an electrode while the cornea approaches the electrode on the opposite side. This change in the orientation of the dipole is reflected as a change in the amplitude and polarity of the EOG signal (Figure 2) so that by registering these changes the movement of the eyeball can be determined. EOG signals have been determined to show amplitudes ranging from 5 to 20 μV per degree of displacement, with a bandwidth between 0 and 50 Hz (Lu et al., 2018).

**Figure 3:**
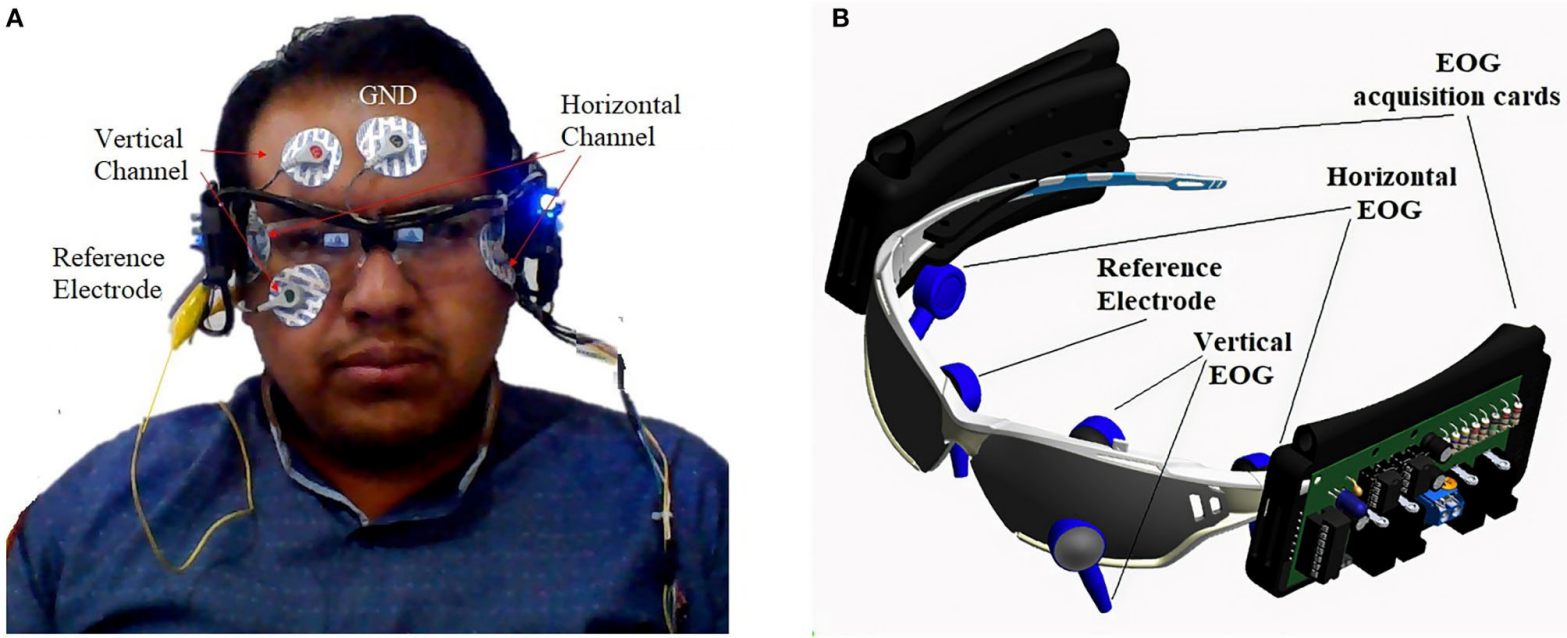
**(A)** Placement of the electrodes. **(B)** Portable EOG signal acquisition and instrumentation system.

The EOG signal is obtained using two pairs of electrodes connected near the eyes, plus a reference electrode on the forehead and another to eliminate muscle noise in the earlobe, thus generating two channels that record horizontal movement and vertical of the eyeball. In total, six silver/silver chloride electrodes are connected (Ag/AgCl), as presented in Figure 3A.

For the acquisition of the reliable EOG signal, an analog processing stage was designed, which includes amplification, isolation, and filtering for each channel (horizontal and vertical) and was complemented by a digital filtering module. The pre-amplification and amplification stage has a 100-dB CMRR, an analog low-pass filter in Butterworth configuration of 40 dB/decade, and a capacitive isolation system for user safety. The designed acquisition system is embedded on a PCB board placed in portable glasses (Figure 3B), to provide security and comfort to the user.

To remove the D.C. level, an integrator circuit is used for feedback of the EOG signal at the reference terminal of the instrumentation amplifier. It acts as a high-pass filter preventing instrumentation amplifiers from being saturated. The muscle signal is considered as noise, and it does not allow obtaining a good interpretation of the EOG signal. To eliminate it, the output of the common-mode circuit of the instrumentation amplifier is connected to the earlobe through an electrode for return noise of the muscle signal at the input of the amplifier, thus subtracting the noise signal of the EOG signal affected by noise. Additionally, the electrode placed on the user's forehead is connected to the isolated ground of the circuit. Through these connections, the D.C. component, generated by involuntary movements and poor electrode connection, is eliminated.

In summary, each type of noise is eliminated and the additive noise is eliminated by means of an integrating circuit that works as a 0.1-Hz high-pass filter that eliminates the DC component that is added to the EOG signal. Impulsive noise caused by muscle movement is eliminated by a common rejection mode circuit connected to the earlobe that is fed back to the instrumentation amplifier in its differential configuration. Due to this property, this noise is subtracted and eliminated. The multiplicative noise is eliminated by means of a second-order digital Notch filter tunable in real time on the device's test platform.

For digital processing of the obtained EOG signal, the horizontal and vertical channels were connected to the differential voltage input of a DAQ6009 acquisition card that communicates with a PC through a USB port of a 25-s sample. The DAQ6009 card is used for the acquisition of the EOG signal because it has a maximum input frequency of 5 MHz; the electrooculography has a bandwidth of DC at 50 Hz, so for the purposes of this work the sample frequency is ideal, complying with the Nyquist sampling theorem. This theorem indicates that the exact reconstruction of a continuous periodic signal from its samples is mathematically possible if the signal is band-limited and the sampling rate is more than double its bandwidth.

**Figure 4:**
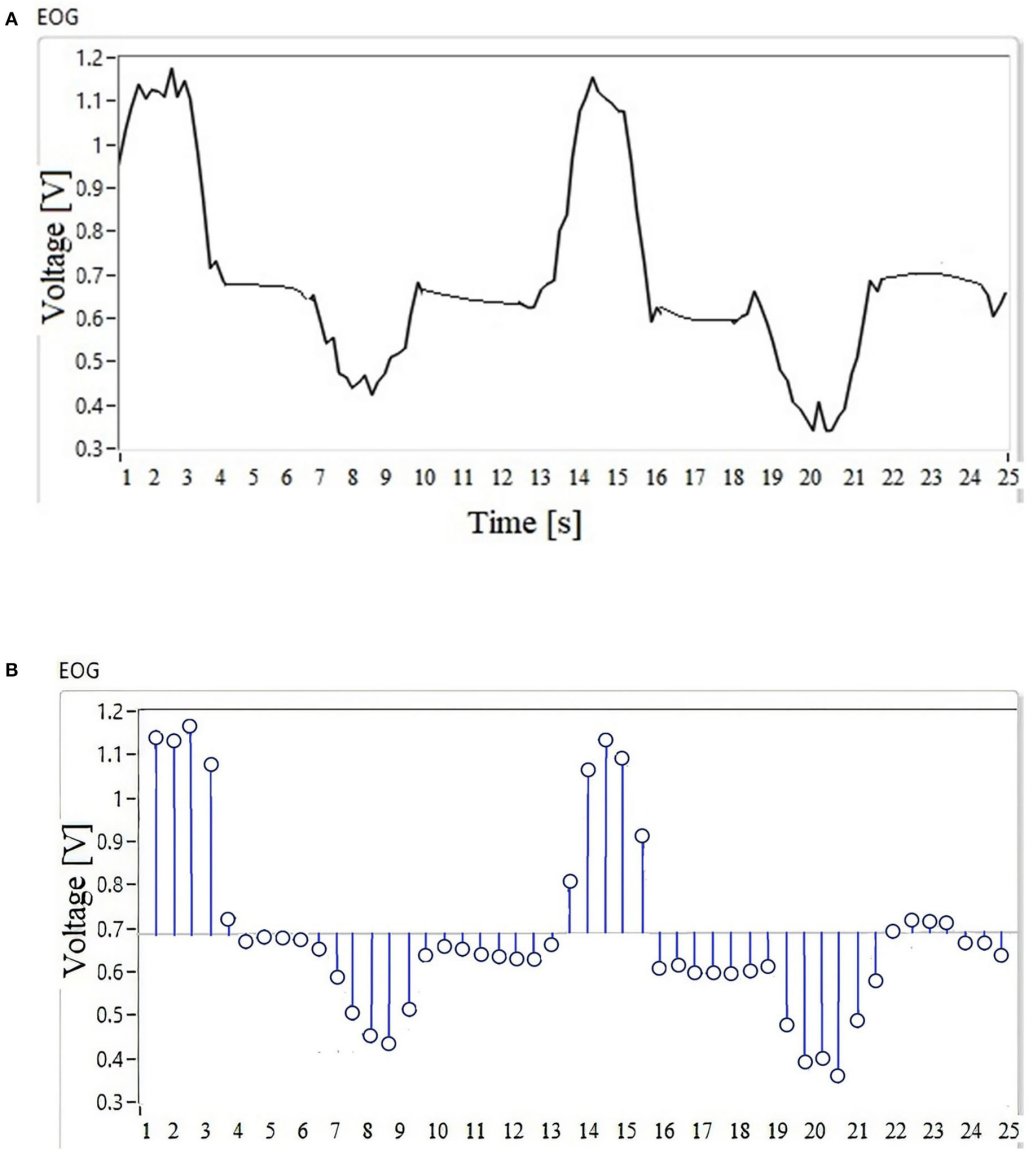
**(A)** Signal waveform. **(B)** Discrete samples.

In Figure 4A, the waveform of the EOG signal of a user is observed when the movement of the gaze to the right and left is performed. This acquisition is done in 25-s time windows generating 48 discrete samples. This signal is digitized by convolution as a function of time with a Dirac pulse train at a frequency of 100 Hz (Equation 1), and the result of signal sampling is presented in Figure 4B.

(1) $$\begin{array}{l} x_{p}\left(t\right)=\sum x\left[nT\right]\delta \left(t-nT\right) \end{array}$$

The EOG signal (Figure 4A) is the input to the fuzzy classifier to generate points according to the workspace of an assistance system that can be a mobile robot, a robotic arm, or a cursor on the screen.

The nature of the EOG signal behavior is non-linear, there is no pattern, and thresholds vary from one individual to another; if this signal is used as input to an HMI system, the classification system must be calibrated for each user or recalibrated if there is a disturbance in the environment. Assistive systems controlled by physiological signals regularly use a database for the system to generate a response to a particular signal; in this type of case, the user must have a training that makes their eye movements generate a signal similar to those stored in the database, thus generating a longer response time in the system. In this case, a database or previous training will not be necessary, because a process of the discrete samples (Figure 4B) of the EOG signal is performed in parallel, which are the input to the MNN designed to perform the interpolation of the discrete data in order to calibrate the system. The objective is to obtain the maximum and minimum values of the voltage threshold; this range is important because it delimits the operation of the fuzzy inference system.

In the next section, the design of the intelligent calibration system is explained first, followed by the operation of the fuzzy inference system.

### Intelligent Calibration System

Due to the need to determine the working threshold of the fuzzy classifier for each person in this section, the modeling of the EOG signal is presented, which allows obtaining the required values of the optimal operating range of the fuzzy inference system. First, the mathematical model of the signal is obtained by means of an MNN; the result of this stage provides an objective function. Then, using genetic algorithms, the voltage thresholds were calculated which, without falling into local values, represent the maximum and minimum values of the signal amplitude when the user guides the gaze. Finally, the custom EOG signal is classified based on its optimal range. This data is sent as the user's optimal thresholds.

#### Multilayer Neural Network

The Widrow–Hoff learning is a training algorithm for an MNN, with the objective of determining synaptic weights; polarization for the classification of data is not linearly separable (An et al., 2020). Given these characteristics, this algorithm was selected for the training of the neural network developed to model the EOG signal.

**Figure 5:**
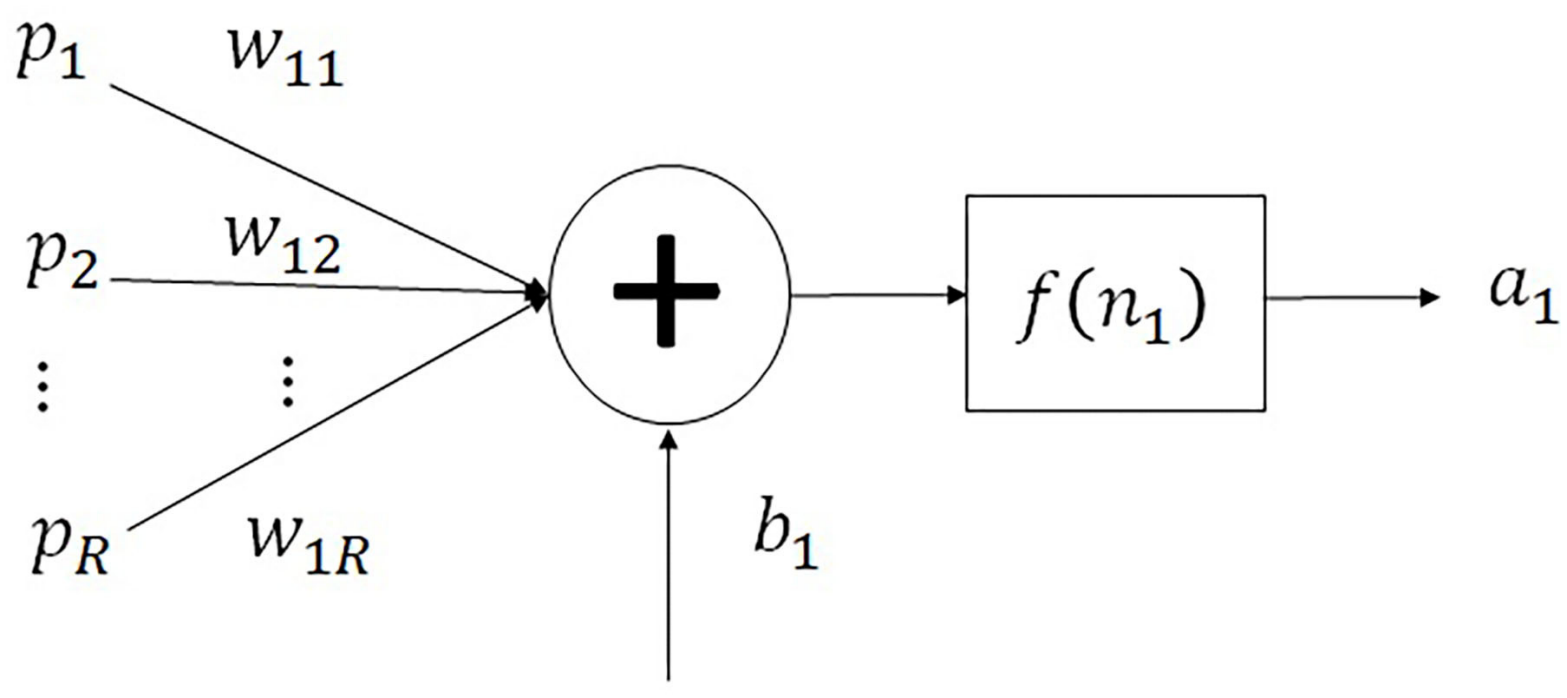
Representation of a monolayer neural network.

In Figure 5, a monolayer neural network is represented, where the vector of the *R* inputs is ***p*** = [*p*^*T*^], $W=\;\left[{W_{SR}}^{T}\right]$ is the synaptic weight matrix, ***b*** = [*b*^*T*^] represents the polarization of the *S* neurons, ***n*** = [*n*^*T*^] represents the net inputs of each of the *S* neurons, and ***a*** = [*a*^*T*^] is the vector of the *S* outputs of the neurons (An et al., 2020).

The output of the monolayer neural network is represented in Equation (2):

(2) $$\begin{array}{l} a=f\left(Wp+b\right) \end{array}$$

The neural network employs activation functions, using a least squares method for its training. The weights are adjusted using the Widrow–Hoff rule to minimize the difference between the output and the objective. This algorithm is an iterative implementation of linear regression, reducing the square error of a linear fit.

A pattern *p*_*q*_ is presented as the input to a network; it responds with an output *a*_*q*_. Due to this, an error vector *e*_*q*_ is formed, which is the subtraction of the desired answer *t*_*q*_, and the neuron's response *a*_*q*_ so that *e*_*q*_ = *t*_*q*_ − *a*_*q*_. Square error is defined as the dot product $e_{q}^{T}e_{q}$ of the error vector that provides the sum of the square errors of each neuron. In order to minimize the square error, the gradient descent is used, whose objective problem is to find *x*_0_ which minimizes function *F*(*x*). In Equation (3), the descending gradient equation is presented to minimize the square error.

(3) $$\begin{array}{l} x_{0}=x_{0}-\alpha \;\frac{dF}{dx}{|}_{x=x_{0}} \end{array}$$

The value of *F*(*x*) is defined as $e_{q}^{T}e_{q}$ whose objective is to minimize the square error by means of an iterative Widrow–Hoff learning. There is a set of test patterns (*p*_*Q*_, *t*_*Q*_), and with these data, the synaptic weights and polarization are found so that the multilayer network responds as desired.

**Figure 6:**
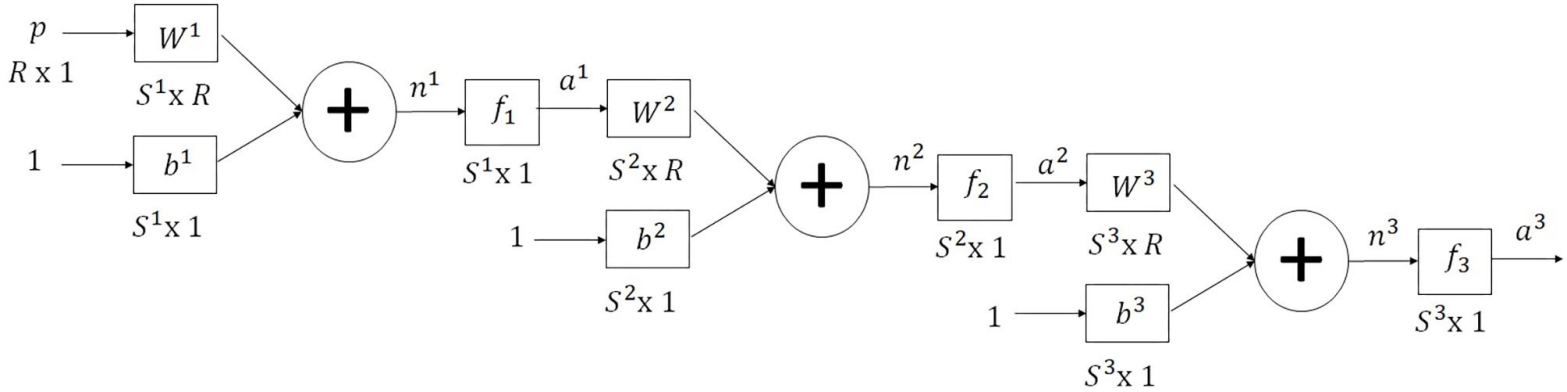
MNN for the calculation of the objective function that describes the behavior of the EOG signal.

The neural network multilayer is implemented to calculate the function that describes the behavior of the EOG signals. It is a neural network that has three layers; it is represented in Figure 6.

The multilayer neural network is used for linear regression; the structure is made up as follows: The input layer has a sigmoid activation function, the hidden layer has a sigmoid activation function, and the output layer has an activation function linear. This is the reason why using non-linear activation functions at the input corresponds to the smooth transition between one sampling point and another, while a linear output activation function allows obtaining numerical values that correspond to the exact value of the sample. In this way, a smooth transition is achieved, and all the sampling points are covered for a correct modeling. The output of the multilayer network takes values that the EOG signal registers which vary according to each person; using a sigmoid function in layer 3 does not allow to reach these values.

The recursive equation that describes the output of the multilayer neural network represented by *a*^*M*^ with input patterns *p* through *q*, for a neural network with *M* layers, is presented in Equation (4), where *X*^*M*^ represents the polarization and synaptic weights of neuron *M*. The solution is more complex because these parameters must be calculated for each of the neurons that make up the multilayer.

(4) $$\begin{array}{l} a^{M}=\;f^{M}(W^{M}f^{M-1}(W^{M-1}\;\cdots \;f^{2}\left(W^{2}f^{1}\left(W^{1}p+b^{1}\right)+b^{2}\right)\\ \;\cdots +b^{M-1})+b^{M})\;\Rightarrow \;\\ a^{m}=f^{m}\left(W^{m}a^{m-1}+b^{m}\right) \end{array}$$

The objective is to minimize the square error, which is a function of *X*^*M*^ arrays (Equation 5).

(5) $$\begin{array}{l} F\left(X^{1},\cdots \;,X^{M}\right)=\;e_{q}^{T}e_{q}\;whereX^{m}={\left[W^{m}\;b^{m}\right]}^{T} \end{array}$$

The function obtained $F\left(X^{1},\cdots \;,X^{M}\right)=\;e_{q}^{T}e_{q}$; the gradient descent method is used to find synaptic weights and polarizations that minimize square error. An optimization method has been obtained that is found when defining the error gradient and is minimized with respect to the parameters of the neural network, as indicated in Equation (6).

(6) $$\begin{array}{r} X^{m}=\;X^{m}-\alpha \;\frac{dF}{dX^{m}}{|}_{x^{m}=x_{0}^{m}}\;\\ X^{m}={\left[W^{m}\;b^{m}\right]}^{T} \end{array}$$

The variation of the mean square error with respect to the synaptic weights and the polarization of the corresponding neuron is described in Equation (7).

(7) $$\begin{array}{l} \frac{dF}{dX^{m}}=\left[\frac{dF}{dx_{1}^{m}}\;\ldots \;\frac{dF}{dx_{S^{m}}^{m}}\right] \end{array}$$

To calculate the gradient $\frac{dF}{dX^{m}}$, the function can be decomposed using the chain rule as the variation of *F* with respect to net input *n*_*i*_, and the product of the variation of the net input with respect to the variation of the neuron's polarization and synaptic weights *i* is represented by *X*_*i*_. The net input is represented as the product of the vectors $n_{i}=x_{i}^{T}z$; the results is seen in Equation (8).

(8) $$\begin{array}{l} \frac{dF}{dx_{i}}=\frac{dn_{i}}{dx_{i}}\frac{dF}{dn_{i}}=zs_{i\;} \end{array}$$

There is variation of function *F*, which is the square error with respect to any net input; in any layer of the neuron, it is represented with an **s** and is called sensitivity. In the sensitivity gradients in Equation (8), the vector *z* is factored and is replaced by the input augmented with 1 in the last element; the input of layer *n* is the output of the previous layer, so $z^{m}=\left[\begin{array}{c} a^{m-1}\\ 1 \end{array}\right]$ and applying the transposed operator the Equation (9) is obtained.

(9) $$\begin{array}{l} \frac{dF}{dX^{m}}={\left[zs_{i}\;\ldots \;zs_{s^{m}}\right]}^{m}=\;z^{m}{\left(s^{m}\right)}^{T}=\left[\begin{array}{c} a^{m-1}\\ 1 \end{array}\right]{\left(s^{m}\right)}^{T}\\ \;=\left[\begin{array}{c} a^{m-1}{\left(s^{m}\right)}^{T}\\ {\left(s^{m}\right)}^{T} \end{array}\right]\;\\ \frac{dF^{T}}{dX^{m}}=\left[s^{m}{\left(a^{m-1}\right)}^{T}\;s^{m}\right] \end{array}$$

If Equation (9) in the formula for the descending gradient of Equation (3) and the vector *X*^*m*^ is replaced in terms of synaptic weights *W* and polarization *b*, Equations (10) and (11) are obtained which determine the iterative method for learning a multilayer neural network by Widrow–Hoff.

(10) $$\begin{array}{l} {\left(X^{m}\right)}^{T}={\left(X^{m}\right)}^{T}-\alpha \frac{dF^{T}}{dX^{m}}\; \end{array}$$

(11) $$\begin{array}{r} W^{m}=\;W^{m}-\alpha s^{m}{\left(a^{m-1}\right)}^{T}\\ b^{m}=b^{m}-\alpha s^{m}\;\\ For\;\forall m\in \left[1,\;\ldots ,\;M\right] \end{array}$$

Now the calculation of the sensitivities must be carried out, which is the basis of the backpropagation algorithm. Sensitivity is defined as the derivative of the function, which is the square error, with respect to the net input of the neuron (Equation 12).

(12) $$\begin{array}{l} \frac{dF}{dn^{m-1}}=\;\frac{dn^{m}}{dn^{m-1}}\frac{dF}{dn^{m}} \end{array}$$

In Equation (12) applying the chain rule, we have the variation of *F* with respect to the net input of the layer *m*, as well as the variation of the net input of the layer *m* with respect to the net input of the previous layer *n*^*m*−1^. If the nomenclature of sensitivities is used, Equation (13) is obtained.

(13) $$\begin{array}{l} s^{m-1}=\frac{dn^{m}}{dn^{m-1}}s^{m} \end{array}$$

Equation (13) indicates the sensitivity of the previous layer *s*^*m*−1^ which is calculated from the sensitivity of the back layer *s*^*m*^. This relationship is what gives it the name of backpropagation because the sensitivity will be propagated from the last layer to the first layer of the neural network to calculate the sensitivity in each one. The net inputs of two consecutive neural networks are related by Equation (14).

(14) $$\begin{array}{l} n^{m}=W^{m}f^{m-1}\left(n^{m-1}\right)+b^{m} \end{array}$$

There is an equation where the net input *n*^*m*^ depends on *f*, which is the activation function of the net input *n*^*m*−1^ from the previous layer. Using the chain rule, the result is expressed in Equation (14), where the derivative of the activation function *f*^*m*−1^ with respect to the net input of the previous layer *n*^*m*−1^ is expressed as *Ḟ*^*m*−1^(*n*^*m*−1^). The second derivative of the net input *n*^*m*^ in relation to the activation function *f*^*m*−1^ is obtained by deriving Equation (13) which results in the transposed vector of the synaptic weights of layer *m* using the equation (*W*^*m*^)^*T*^, where Equation (15) is obtained.

(15) $$\begin{array}{r} \frac{dn^{m}}{dn^{m-1}}=\;\frac{df^{m-1}}{dn^{m-1}}\frac{dn^{m}}{df^{m-1}}=\;{Ḟ}^{m-1}\left(n^{m-1}\right){\left(W^{m}\right)}^{T}\;\\ s^{m-1}=\;{Ḟ}^{m-1}\left(n^{m-1}\right){\left(W^{m}\right)}^{T}s^{m}\;\\ \text{For}\forall m\;\in \left[M,\ldots ,\;2\right] \end{array}$$

Equation (15) calculates each of the sensitivities; in order to carry out this process, it is necessary to calculate the sensitivity of the last layer *M* (where *s*^*m*^ = *s*^*M*^). Applying the chain rule to deduce the sensitivity *s*^*M*^, the last layer of the sensitivity definition is known to be the derivative of the objective function to be minimized with respect to the net input of the last layer *n*^*m*^, *F* is the square error $F=e_{q}^{T}e_{q}$, and the error is the difference of the desired response *t*_*q*_ and the response of the last layer, defined as the activation function *f*^*M*^ (*n*^*M*^), that is, $e_{q}=\;t_{q}-f^{M}\left(n^{M}\right)$; the result of the said process is observed in Equation (16).

(16) $$\begin{array}{l} s^{M}=\frac{dF}{dn^{M}}=\frac{df^{M}}{dn^{M}}\;\frac{de_{q}}{df^{M}}\frac{dF}{de_{q}}=\frac{df^{M}}{dn^{M}}\left(-1\right)\left(2e_{q}\right)\\ \;=-2{Ḟ}^{M}\left(n^{M}\right)e_{q}\; \end{array}$$

(17) $$\begin{array}{l} s^{M}=-2{Ḟ}^{M}\left(n^{M}\right)e_{q} \end{array}$$

The definition of *Ḟ*^*M*^ (*n*^*M*^) which is the derivative of the activation functions with respect to the net input; this process is represented in Equation (17). The derivative generates a matrix containing the gradients of each of the activation functions of the neural network with respect to its net input, and so on, until it reaches the last neuron.

(18) $$\begin{array}{l} {Ḟ}^{M}\left(n^{M}\right)\;={Ḟ}^{m}\left(n^{m}\right)\;\frac{df^{m}}{dn^{m}}=\;\left[\frac{df_{1}^{m}}{dn^{m}}\;\ldots \;\frac{df_{s^{m}}^{m}}{dn^{m}}\right]\\ \;=\;\left[\begin{array}{cccc} \frac{df_{1}^{m}}{dn_{1}^{m}}\; & 0\; & \cdots & 0\\ 0\; & \frac{df_{2}^{m}}{dn_{2}^{m}}\; & \cdots & 0\\ \vdots & \vdots & \ddots & \vdots \\ 0 & 0 & \ldots & \frac{df_{s^{m}}^{m}}{dn_{s^{m}}^{m}} \end{array}\right]=diag\;\left(\frac{df_{1}^{m}}{dn_{1}^{m}}\right) \end{array}$$

The derivative must exist for any value of the net input that is a continuous function, and there are three activation functions to which the operation *Ḟ*^*M*^ (*n*^*M*^) must be calculated. The MNN is made up of three neurons, but two activation functions are used: the sigmoidal and the linear. The result of implementing Equation (17) in the activation functions of the neural network is presented in Equation (18) for the sigmoid activation function and in Equation (19) for the linear activation function.

• Logistics sigmoid.

$$\begin{array}{l} a_{i}=f_{i}\left(n_{i}\right)=\;\frac{1}{1+e^{-ni}}\to \;\frac{df_{i}}{dn_{i}}=\left(1-a_{i}\right)a_{i}\; \end{array}$$

If all neurons have the same function:

(19) $$\begin{array}{l} {Ḟ}^{m}\left(n^{m}\right)=diag\;\left(\left(1-a_{i}^{m}\right)a_{i}^{m}\right) \end{array}$$

• Lineal function.

$$\begin{array}{l} a_{i}=f_{i}\left(n_{i}\right)=\;ni\to \;\frac{df_{i}}{dn_{i}}=1\; \end{array}$$

If all neurons have the same function:

(20) $$\begin{array}{l} {Ḟ}^{m}\left(n^{m}\right)=1 \end{array}$$

The descending backpropagation algorithm for calculating an objective function that models the behavior of the EOG signal by discrete samples is presented in **Listing 1**.

**Listings 1:** Backpropagation algorithm for interpolation of an EOG signal using a multilayer neural network, through discrete samples.

Pseudocode: algorithm for interpolation of an EOG
Random initialization of *W*^*m*^ and *b*^*m*^ for ∀*m*
Since epochs = *1* to *N*_*epochs*_ repeat
Since *q* = 1 to *Q* repeat (*Q* = *Sample vector size*)
1. Forward propagation.
*a*^*m*^ = *f*^*m*^(*W*^*m*^*a*^*m*−1^ + *b*^*m*^) for ∀*m*
2. Backpropagation.
$e_{q}=t_{q}-a_{0}^{M}$
$s^{M}=-2{Ḟ}^{M}\left(n^{M}\right)e_{q}$
*s*^*m*−1^ = *Ḟ*^*m*−1^(*n*^*m*−1^)(*W*^*m*^)^*T*^ *s*^*m*^ for ∀*m* ∈ [*M*, …, 2]
3. Update for ∀ *m*
*W*^*m*^ = *W*^*m*^ − α*s*^*m*^(*a*^*m*−1^)^*T*^
*b*^*m*^ = *b*^*m*^ − α*s*^*m*^
End
End

**Figure 7:**
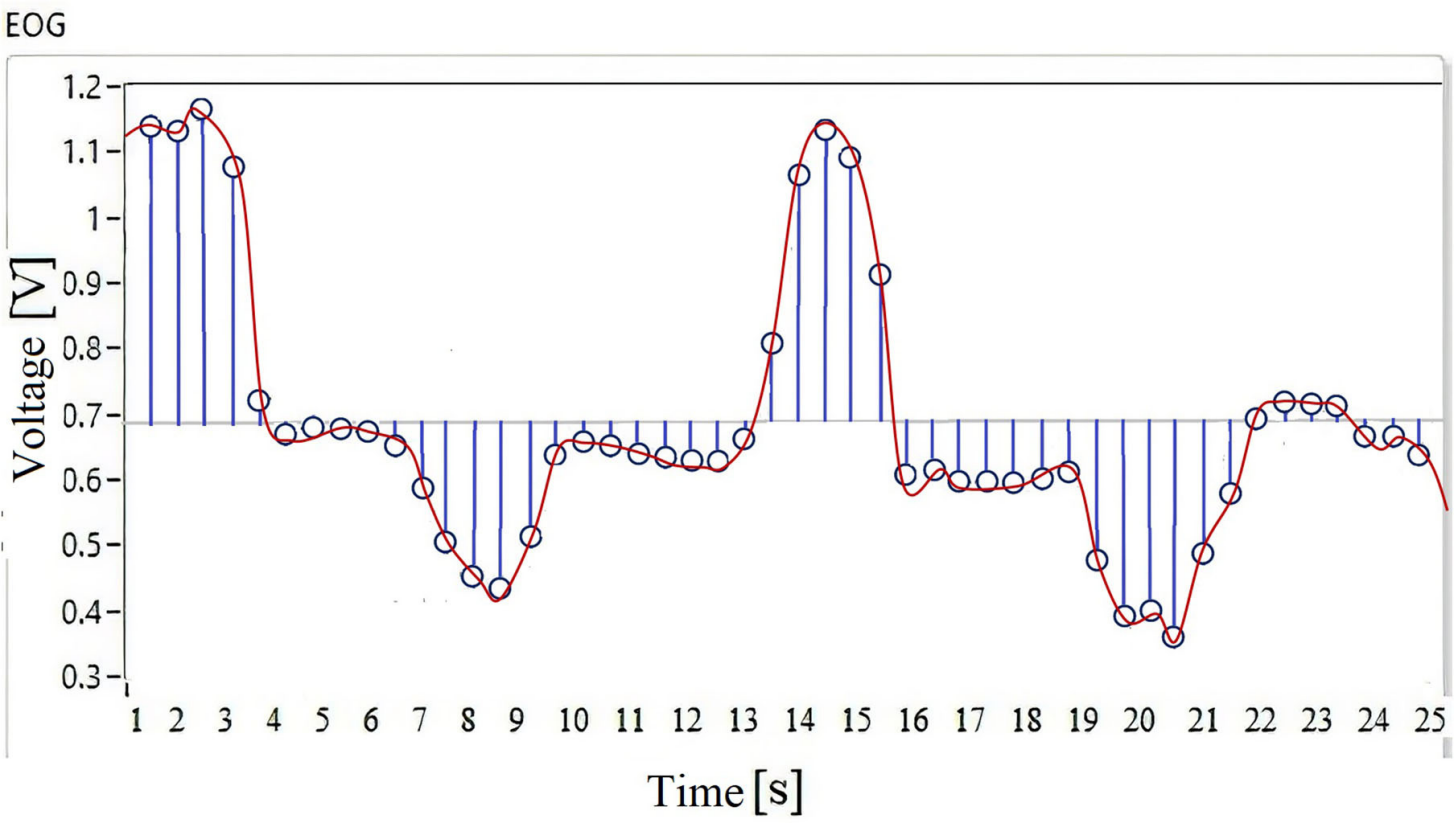
Interpolation using a multilayer neural network, to calculate an objective function to be optimized.

From the acquisition of the EOG signal, 48 discrete samples are obtained that are stored as a data vector *Q*; the non-linear regression is applied on these data. The algorithm calculates a function that passes through all the discrete points. Figure 7 shows a trend line resulting from the neural network when interpolating the signal samples, this being the output of the last layer.

The function *f*(*x*) = *a*^*m*^ = *f*^*m*^(*W*^*m*^*x*^*m*−1^ + *b*^*m*^) describes the behavior of the EOG signal of each individual, depending on the variability of the value of the synaptic weights *W*^*m*^ and polarization *b*^*m*^. Through this method, the analytical description of the physiological signal is obtained.

By having a mathematical function that describes the individual characteristics of each user, information is obtained that allows a classification system to adapt to the variability that physiological signals present. As can be seen in Figure 7, this signal has several positive and negative data on a threshold; in order to determine the operating range of a system, it is necessary to know the amplitude in which the signal varies for each user. The objective is to record the maximum and the minimum value of the signal threshold to calibrate the fuzzy inference system.

#### Genetic Algorithm

An optimization problem can be formulated as a process where the optimal value *x* which minimizes or maximizes the objective function is found. In this case, the objective function is the result from interpolation performed with a multilayer neural network and it is determined by Equation (20). Considering that *W*^*m*^ represents the value of the synaptic weights, *b*^*m*^ is the value of polarization of each layer, *m* is the maximum number of layers, and **X** represents the vector of decision variables. **X** represents the candidate solution set, also known as the search space or solution space, such that **x** ∈ **X**. The search space is limited by the lower (*l*) or upper (*u*) limits of each of the *d* variables, as indicated in Equation (20).

(21) $$\begin{array}{r} f\left(x\right)=f^{m}\left(W^{m}x^{m-1}+b^{m}\right)\;\in \;\mathbb{R},\\ X=\left\{x\;\in \;\mathbb{R}\;|\;l_{i}\;\leq \;x_{i}\;\leq \;u_{i},\;d=1\right\} \end{array}$$

The objective function obtained is a complex problem to solve using classical optimization methods, because it contains a set of local optimums. Therefore, an evolutionary method like genetic algorithms is a good alternative for its solution. When the mathematical model of the EOG signal is obtained as a result of the processing of the neural network, it is represented as a mathematical function with local positive and negative thresholds; using classical optimization techniques, it is not possible to determine the range of the signal because it presents different ridges and valleys of different amplitudes, so the two objectives sought are to obtain the maximum and minimum optima regardless of the variable characteristic of the signal. For the maximum optimal value, the 15 iterations of the genetic algorithm are applied, and to obtain the minimum optimal value, a negative sign multiplies the objective function and the 15 iterations of the genetic algorithm are performed again.

For genetic algorithms (GA), each candidate solution is considered an individual that belongs to a population, and its level of “adaptation” is the value obtained when evaluating each of the candidate solutions with the objective function (Leardi, 2003). Basically, a GA is an algorithm that generates a random population of parents; during each generation, it selects pairs of parents considering their value *f*(*x*) and exchanges of genetic material or crosses are made to generate pairs of children; such children will be mutated with a certain probability and will ultimately compete to survive the next generation with the parents, a process known as elitism.

The algorithm corresponding to a GA is indicated below:

Number of dimensions d = 1

Search space limits l = 0 y u = 25

Number of iterations Niter = 15

Population size Np = 48

Number of bits per dimension Nb = 11

Initialization by the equation: $x_{n}=\;l+rand{\left(.\right)}^{*}\left(u-l\;\right)$

Selection of parents (Roulette Method): Each individual is evaluated considering the objective function.

The cumulative of the objective function is calculated as *E*, as indicated in Equation (21).

(22) $$\begin{array}{l} E=\overset{Nb}{\underset{i=1}{\sum }}f\left(x_{i}\right) \end{array}$$

The possibility of selection of each individual is calculated, as shown in Equation (22).

(23) $$\begin{array}{l} p_{i}=\frac{f\left(x_{i}\right)}{E} \end{array}$$

The cumulative probability of each individual is calculated, represented in Equation (23).

(24) $$\begin{array}{l} q_{i}=\overset{i}{\underset{j=1}{\sum }}p_{j} \end{array}$$

Then, the selection of the parents is made:

A uniformly distributed random number is generated.

The parent that satisfies the condition *q*_*i*_ > *r* is selected, where *r* is a random value between 0 and 1.

In a GA, it is necessary to determine certain parameters for its design; these are as follows.

*Cross*: It consists of randomly generating a location within each individual which will serve as a reference for the exchange of genetic information, previously converting to binary values. We consider a parent pair of 11 binary data and an initial cross point with a value of 7 generated randomly. Each individual is divided into two parts: one of 5 bits and the other of 6 bits; later, the complementary parts of each individual will be united, to form the descendants.
*Mutation*: The individuals in the population are made up of binary chains; the mutation is carried out by changing with some probability the bits of each descendant individual, generating a population of mutated children, although it is also necessary to convert the said population to real numbers in order to evaluate them in the objective function.
*Selection of the fittest:* It is necessary to select the fittest individuals who will survive the next generation. This is achieved through the competition of the parents with the children that were generated after applying the cross and mutation operators; in the case of the binary AG, the original populations of parents are simply mixed, and that of the children generated.

**Figure 8:**
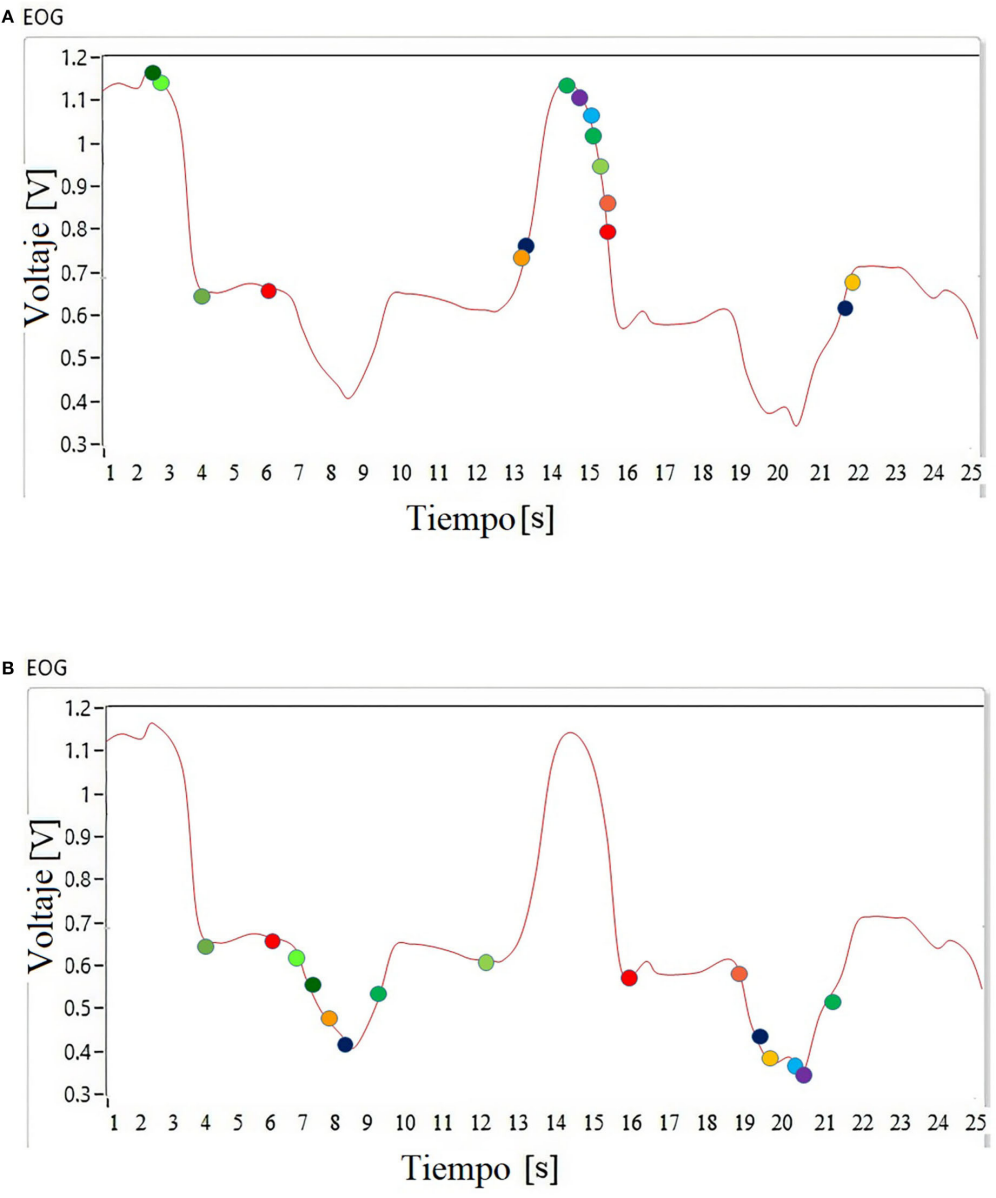
**(A)** Genetic algorithm at optimal maximum. **(B)** Genetic algorithm at optimal minimum.

In Figure 8A, the data is observed in each period of the genetic algorithm cycle in the case of obtaining the optimal maximum, while in Figure 8B the data is observed in the same period of the genetic algorithm, but calculating the optimal minimum.

Table 1 records the data in each period in which the optimization algorithm is evaluated. As an example for a specific user, the data in Table 1 are obtained.

**Table 1:** Values delivered by the genetic algorithm in each period.

**Iteration number**	**Maximum optimum**	**Minimum optimum**
1	0.7328	0.6983
2	0.8102	0.6452
3	0.8392	0.6012
4	0.8583	0.5932
5	0.8873	0.5874
6	0.8921	0.5320
7	0.9012	0.4943
8	0.9058	0.4832
8	0.9134	0.48032
10	0.9239	0.4786
11	0.9323	0.4632
12	0.9532	0.4324
13	0.9832	0.4132
14	1.0983	0.3932
15	1.1193	0.3172

*The values highlighted in gray in Table 1 provide the range to which the EOG classifier should be calibrated for this user, thus assigning to each voltage threshold a coordinate in Cartesian space using fuzzy logic*.

### Fuzzy Inference System

To characterize the EOG signal and to be able to use it to generate Cartesian coordinates, a classification system with fuzzy logic was implemented. This method uses a set of mathematical principles based on degrees of belonging and is performed based on linguistic rules that approximate a mathematical function. The input is the signals of the two previously calibrated EOG horizontal and vertical channels, and the output of the system are Cartesian coordinates within the working space of an assistance system, in this case an anthropomorphic manipulator robot.

The Mamdani-type inference method was implemented to design the fuzzy classifier because it allows the intuitive relationship through syntactic rules between the workspace and the voltage thresholds; this feature is very useful when generating a point in Cartesian space in real time.

In the mathematical interpretation of Mamdani's fuzzy controller, there are two fuzzy antecedents expressed by membership functions of the linguistic variables *A*′ and *B*′, with a first premise or a valid fact: If *x* is *A*′ and *y* is *B*′, then we have a set of fuzzy rules expressed in the form; if *x* is *A*_*i*_ and *y* is *B*_*i*_ then *z* is *C*_*i*_. Where *x* is the voltage range of the calibrated EOG signal for the input or the robot workspace for the output, *A*′ and *B*′ are the antecedents of the linguistic variables expressed by membership functions and *C*_*i*_ is the consequent of a fuzzy set *z*. In the end, when evaluating all the fuzzy rules, we have a conclusion set *z* which is *C*′; this approach is represented in Equation (24) using the Mamdani inference model.

(25) $$\begin{array}{l} \frac{\begin{array}{ll} x\text{ is }A^{\prime }\text{ and }y\text{ is }B^{\prime } & \text{ True Premise}\\ \text{ If }x\text{ is }A_{1\;}\text{ and }y\text{ is }B_{1},\text{ then }z\text{ is }C_{1} & \text{ Fuzzy rule }1\\ \text{ If }x\text{ is }A_{2\;}\text{ and }y\text{ is }B_{2},\text{ then }z\text{ is }C_{2} & \text{ Fuzzy rule }2\\ \;\vdots & \;\\ \text{ If }x\text{ is }A_{i\;}\text{ and }y\text{ is }B_{i},\text{ then }z\text{ is }C_{i} & \text{Fuzzy rule i} \end{array}}{z\text{ is }C^{\prime }\text{ Set Conclusion}.} \end{array}$$

**Figure 9:**
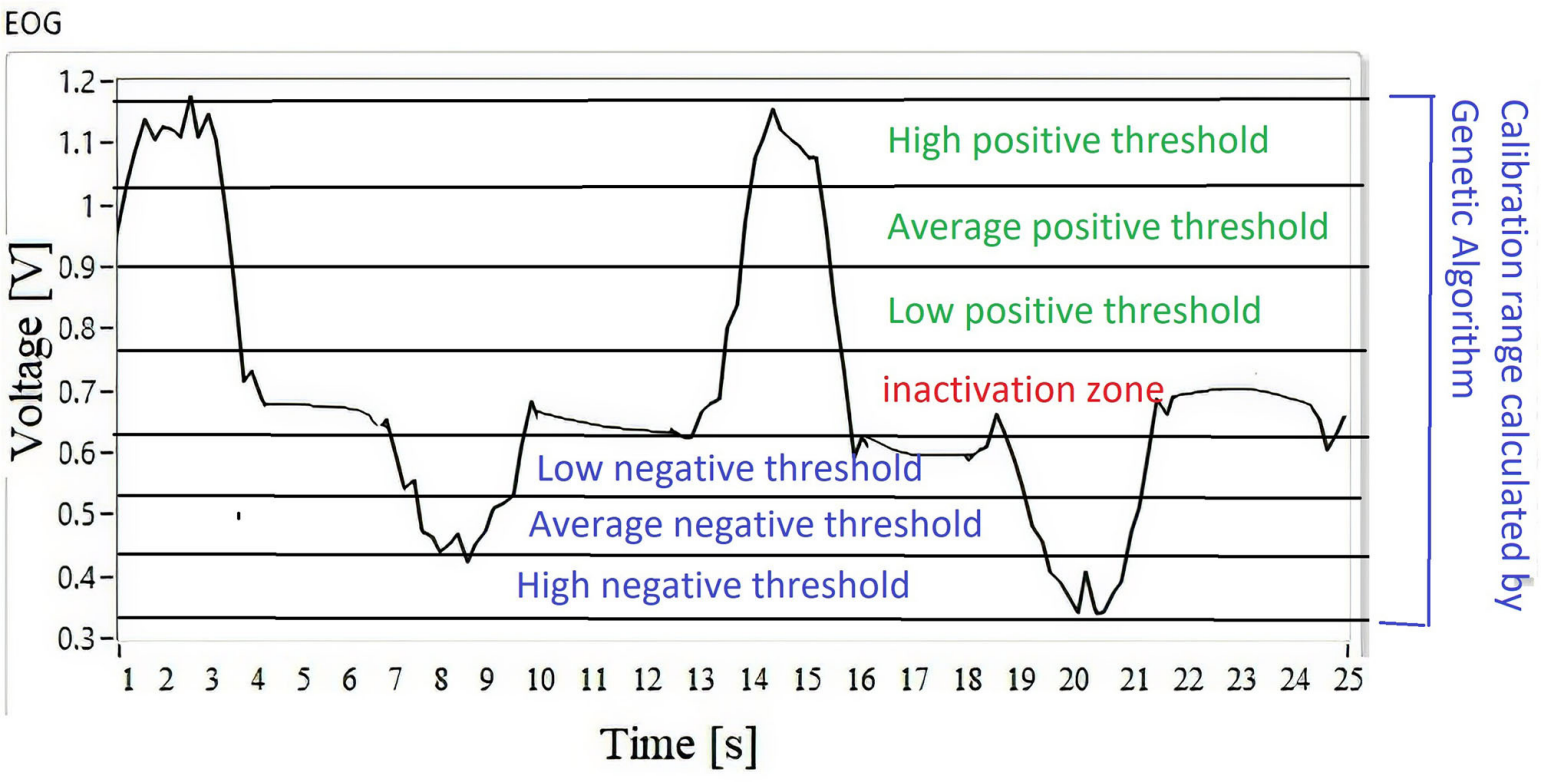
Classification using thresholds of the EOG signal.

By classifying the EOG signal by thresholds from positive to negative, leaving an inactivation zone as indicated in Figure 9, the response relationship is performed in the Cartesian space of the anthropomorphic robot. The entire workspace is mapped according to the threshold registered in the classifier.

The membership function used to verify the performance of the fuzzy classifier is a Gaussian-type function, such as that presented in Equation (25). The implemented membership functions are Gaussian, because the transition between one membership function to another is smooth; it also helps to generate trajectories from one point to another without using cubic polynomials like the Spline technique.

(26) $$\begin{array}{l} \text{Gaussian }:f\left(x;a,b\right)=\;\{e^{{\left(\frac{x-a}{b}\right)}^{2}} \end{array}$$

where *a* defines the mean value of the Gaussian bell, while *b* determines how narrow the bell is.

**Figure 10:**
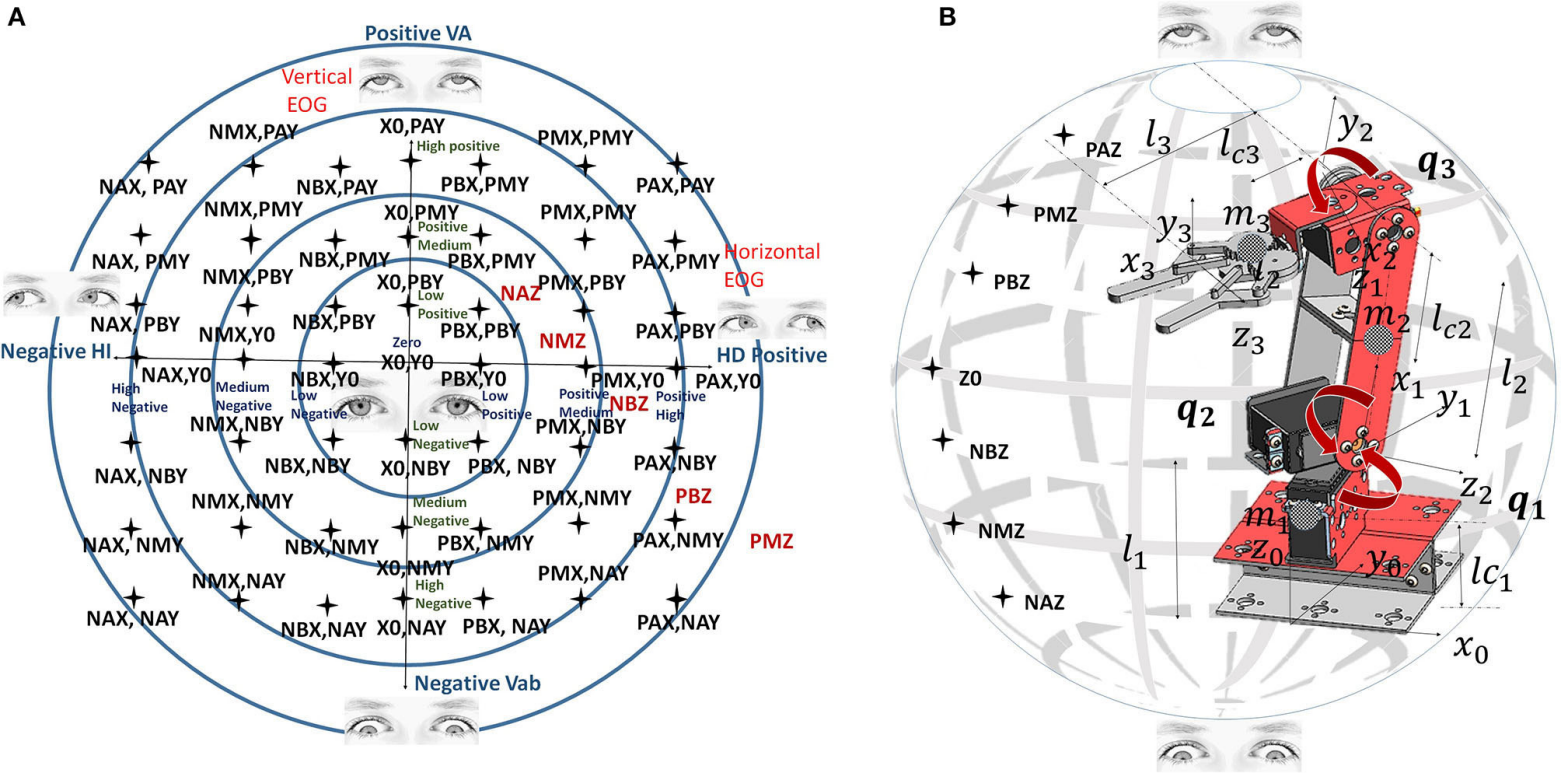
**(A)** Relation of the robot workspace and the EOG signals. **(B)** Robot workspace and dimensions.

The output is the work space of the anthropomorphic robot, which is represented as a hollow sphere; previously, studies of direct and inverse kinematics were performed to calculate its work space, as well as robot dynamics to apply control algorithms for monitoring of the paths generated by the fuzzy classifier. The robot with which experimental tests were carried out is presented in Figure 10B.

According to the voltage level that each linguistic variable represents, the inputs are defined, *x* = EOG vertical/EOG horizontal, for fuzzy classifier inputs that use Gaussian membership functions *T*(*x*); the names of these functions are presented in **Algorithm 1**.

**Algorithm 1:** Name of each of the Classifier input Functions.

*T*(*x*) = Negative Vertical/Horizontal High *(NAV/NAH)*, Vertical/Horizontal Medium Negative (NMV/NMH) Vertical/Horizontal Low Negative (NBV/NBH) Vertical/Horizontal Zero (ZV/ZH), Positive Vertical/Horizontal Low (PBV/PBH), Positive Vertical/Horizontal Medium (PMV/PMH), Vertical/Horizontal High Positive (PAV/PAH).

According to the voltage level represented by each linguistic variable, the workspace is defined with *y* = Position *X*/*Y*/*Z*, for the outputs of the fuzzy classifier, using the Gaussian membership functions *T*(*y*). The name of the functions is indicated in **Algorithm 2**.

**Algorithm 2:** Name of each of the Output Functions of the Classifier.

*T*(*y*) = Negative High X/Y/Z (NAX/NAY/NAZ) Negative Medium X/Y/Z (NMX/NMY/NMZ) Negative Low X/Y/Z (NBX/NBY/NBZ) Zero X/Y/Z (X0/Y0/Z0) Positive Low X/Y/Z (PBX/PBY/PBZ) Positive Medium X/Y/Z (PMX/PMY/PMZ) Positive High X/Y/Z (PAX/PAY/PAZ).

**Algorithm 3** establishes the range of the membership functions of the optimization of the modeling of the EOG signal from the results obtained in Table 1, corresponding to each vertical and horizontal channel modeled by Gaussian membership functions.

**Algorithm 3:** Input membership functions.

**x=EOG Vertical**	**x=EOG Horizontal**
**EOG input signal:** [0.3172 (*optimal minimum*) *to* 1.1923 (*optimal maximum*)] *Volts*. Peak-to-peak voltage of an EOG signal (Optimized Variable Rating Range).	
*M*(*NAV*) = *Gaussian* (*x*, [0.31 0.31 0.4 0.49])	*M*(*NAH*) = *Gaussian* (*x*, [0.31 0.31 0.4 0.49])
*M*(*NMV*) = *Gaussian* (*x*, [0.4 0.49 0.58 0.67])	*M*(*NMH*) = *Gaussian* (*x*, [0.4 0.49 0.58 0.67])
*M*(*NBV*) = *Gaussian* (*x*, [0.49 0.58 0.67 0.76])	*M*(*NBH*) = *Gaussian* (*x*, [0.49 0.58 0.67 0.76])
*M*(*ZV*) = *Gaussian* (*x*, [0.67 0.76 0.85 0.94])	*M*(*ZH*) = *Gaussian* (*x*, [0.67 0.76 0.85 0.94])
*M*(*PBV*) = *Gaussian* (*x*, [0.76 0.85 0.94 1.03])	*M*(*PBH*) = *Gaussian* (*x*, [0.76 0.85 0.94 1.03])
*M*(*PMV*) = *Gaussian* (*x*, [0.85 0, 94 1.03 1.10])	*M*(*PMH*) = *Gaussian* (*x*, [0.85 0, 94 1.03 1.10])
*M*(*PAV*) = *Gaussian* (*x*.[1.03 1.10 1. 19 1.19])	*M*(*PAH*) = *Gaussian* (*x*.[1.03 1.10 1. 19 1.19])

**Algorithm 4** establishes the range of the membership functions of the fuzzy classifier outputs for each of the Cartesian coordinates in *f*(*p*_*x*_, *p*_*y*_, *p*_*z*_); the Gaussian membership functions are used depending on the workspace of any assistance system, whose positions are expressed in Cartesian coordinates, in this case that of an anthropomorphic robot with three degrees of freedom.

**Algorithm 4:** Output membership functions.

**x=Position p_x_**	**x=Position p_y_**	**x=Position p_z_**
**Robot workspace:** [−30 *a* 30] *cm* Distance in centimeters within the workspace (this range may vary depending on the device workspace).		
*M*(*NAX*) = *Gaussian* (*x*, [− 30 − 30 − 22.5 − 15])	*M*(*NAY*) = *Gaussian* (*x*, [− 30 − 30 − 22.5 − 15])	*M*(*NAZ*) = *Gaussian* (*x*, [− 30 − 30 − 22.5 − 15])
*M*(*NMX*) = *Gaussian* (*x*, [− 22.5 − 18.7 − 11.2 − 7.5])	*M*(*NMY*) = *Gaussian* (*x*, [− 22.5 − 18.7 − 11.2 − 7.5])	*M*(*NMZ*) = *Gaussian* (*x*, [− 22.5 − 18.7 − 11.2 − 7.5])
*M*(*NBX*) = *Gaussian* (*x*, [− 15 − 11.2 − 3.7 0])	*M*(*NBY*) = *Gaussian* (*x*, [− 15 − 11.2 − 3.7 0])	*M*(*NBZ*) = *Gaussian* (*x*, [− 15 − 11.2 − 3.7 0])
*M*(*X*0) = *Gaussian* (*x*, [− 7.5 − 3.7 3.7 7.5])	*M*(*Y*0) = *Gaussian* (*x*, [− 7.5 − 3.7 3.7 7.5])	*M*(*Z*0) = *Gaussian* (*x*, [− 7.5 − 3.7 3.7 7.5])
*M*(*PBX*) = *Gaussian* (*x*, [0 3.7 11.2 1.5])	*M*(*PBY*) = *Gaussian* (*x*, [0 3.7 11.2 1.5])	*M*(*PBZ*) = *Gaussian* (*x*, [0 3.7 11.2 1.5])
*M*(*PMX*) = *Gaussian* (*x*, [7.5 11.2 18.7 22.5]	*M*(*PMY*) = *Gaussian* (*x*, [7.5 11.2 18.7 22.5]	*M*(*PMZ*) = *Gaussian* (*x*, [7.5 11.2 18.7 22.5]
*M*(*PAX*) = *Gaussian* (*x*, [15 22.5 30 30])	*M*(*PAY*) = *Gaussian* (*x*, [15 22.5 30 30])	*M*(*PAZ*) = *Gaussian* (*x*, [15 22.5 30 30])

The relationship between the voltage thresholds and the robot workspace is indicated by 113 inference rules. The workspace is classified on dividing surfaces by slices from the positive to the negative threshold; the horizontal EOG is related to the positions on the *x*-axis, while the vertical EOG is related to the positions on the *z*-axis and *y*-axis. By having the membership functions for the inputs and outputs of the fuzzy classifier, syntactic rules are implemented that indicate the points that form the trajectory to be followed by the manipulator robot. Figure 10A represents the relationship of the workspace in the *XY* plane by means of a Cartesian axis; the horizontal EOG channel is represented by the abscissa, and the vertical EOG channel is represented by the ordinates. Each of the concentric circles represents a layer of the plane that encompasses the position correlation in the *XY* plane and the EOG signal voltage threshold value from positive values above the baseline and values below this reference which take negative values. Figure 10B represents the relationship of the workspace on the *z*-axis with respect to the vertical EOG channel and the three degrees of freedom robot (*q*_1_, *q*_2_ *and q*_3_).

**Figure 11:**
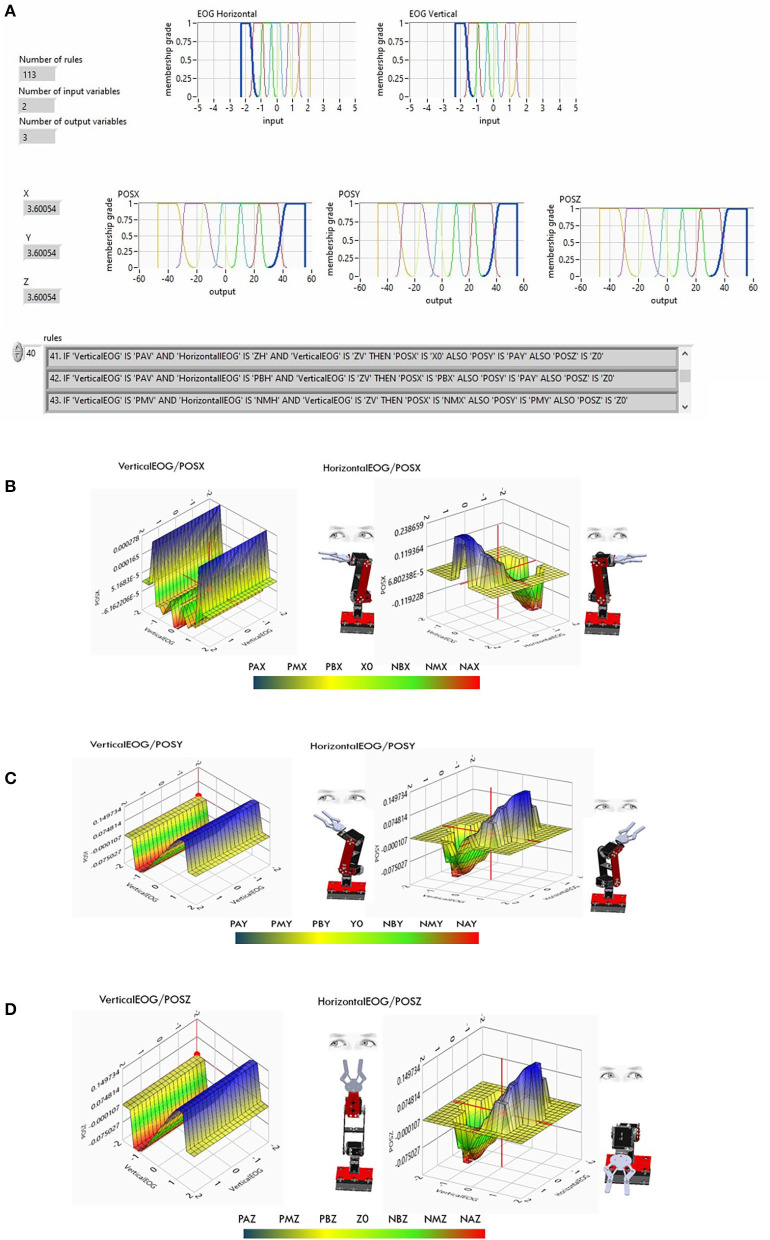
**(A)** Fuzzy classifier of the EOG signal using the 113 inference rules previously calibrated with genetic algorithms. **(B)** *X*-axis control surface. **(C)** *Y*-axis control surface. **(D)** *Z*-axis control surface.

All fuzzy syntactic rules for generating positions through eye movement interaction were introduced into the LabVIEW Design Manager (see Figure 11A) V2019. The horizontal EOG signal corresponds to the *x*-axis or abscissa, while the vertical EOG corresponds to the *y*-axis or ordered y for the *z* coordinates, generating a trajectory in the Cartesian space using a function *f*(*x, y, z*). The LabVIEW V.2019 Design Manager presents the control surfaces for each *X*, *Y*, and *Z* position output relative to the horizontal and vertical channel input data. In each trend of the surfaces, it is observed that while the voltage value in the horizontal/vertical EOG is positive, the graph has a blue color. The direction of position in each of the axes is explained; the graph indicates the positions in *X* to the right (see Figure 11A), in *Y* it indicates the position upward on the *XY* plane (see Figure 11B), and in *Z* the robot's position is higher than the *XY* plane (see Figure 11C). In contrast, if the graph tends toward negative values, it has a red color tone; the position values in *X* are to the left (see Figure 11A), in *Y* it indicates the position down on the *XY* plane (see Figure 11B), and in *Z* the robot position is lower than the *XY* plane (see Figure 11C), covering the entire workspace.

The classifier has the property of being variable in the input membership functions to be adaptive to any user, while the output membership functions are variable in order to adjust the classifier to any navigation system with coordinates in the Cartesian space. This system can be adapted to the generation of trajectories for autonomous aerial vehicles, a pointer for a personal computer in order to write letterforms and for home automation systems; however, for the purposes of this work the fuzzy output classifier adapts to the workspace of an anthropomorphic robot. Table 2 describes the relationship of the horizontal and vertical EOG signals and the robot workspace represented as a hollow sphere. The position is determined by the membership functions of the semantic rules (*p*_*x*_, *p*_*y*_, *and p*_*z*_). The coordinates of the manipulator robot are previously defined for each value of the acquisition potential of the EOG signal, covering the entire workspace of the robot. For example, the horizontal EOG input is defined in the membership function ZH, the vertical EOG input is defined in the membership function ZV, and the output values are in Cartesian coordinates; they are delimited by the membership functions *X*0, *Y*0, and *Z*0 set as the robot home position.

**Table 2:**
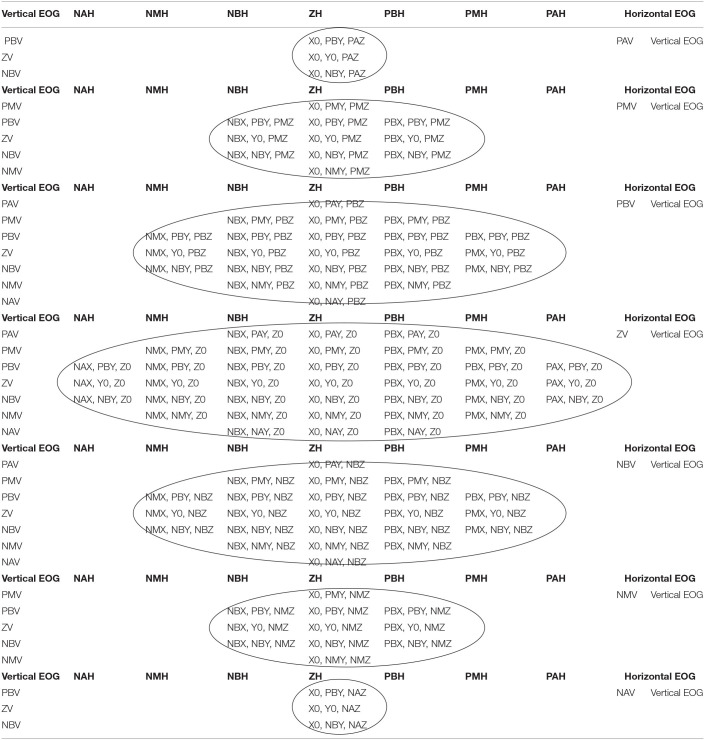
Correspondence of the ocular displacement and the workspace of an anthropomorphic robot with three degrees of freedom.

### Robot Position Control Scheme

The result of the classifier provides the position of the robot in Cartesian coordinates (*X, Y, Z*); to convert these results into desired joint coordinates (*q*_1*d*_, *q*_2*d*_, *q*_3*d*_), the inverse kinematics of the robot are used. These values are the input of the control PD+ algorithm that orders the robot to path tracking.

The control law is expressed in Equation (26).

(27) $$\begin{array}{l} \tau_{PD+}=K_{p}\tilde{q}+K_{v}\dot{\tilde{q}}+M\left(q\right)\ddot{q_{d}}+C\left(q,\dot{q}\right)\dot{q_{d}}+B\dot{q_{d}}+g\left(q\right) \end{array}$$

This algorithm requires the dynamic robot model, so *M*(*q*) is a positive defined symmetric matrix *n x n* which corresponds to the robot's inertia matrix, $C\left(q,\dot{q}\right)$ is an array of *n x n* which corresponds to the matrix of centrifugal forces or Coriolis, *B* is a vector *n x* 1 which determines the viscous friction coefficients, *g*(*q*) is a vector *n x* 1 representing the effect of gravitational force, τ is a vector *n x* 1 indicating torque applied to joint actuators, *K*_*p*_ and *K*_*v*_ are the proportional and derivative constants of the controller, $\tilde{q}$ is joint position error, $\dot{\tilde{q}}$ is the joint speed error, $\ddot{q_{d}}$ is the desired joint acceleration, and $\dot{q_{d}}\;$is the desired joint speed.

## Experiments and Results Analysis

**Figure 12:**
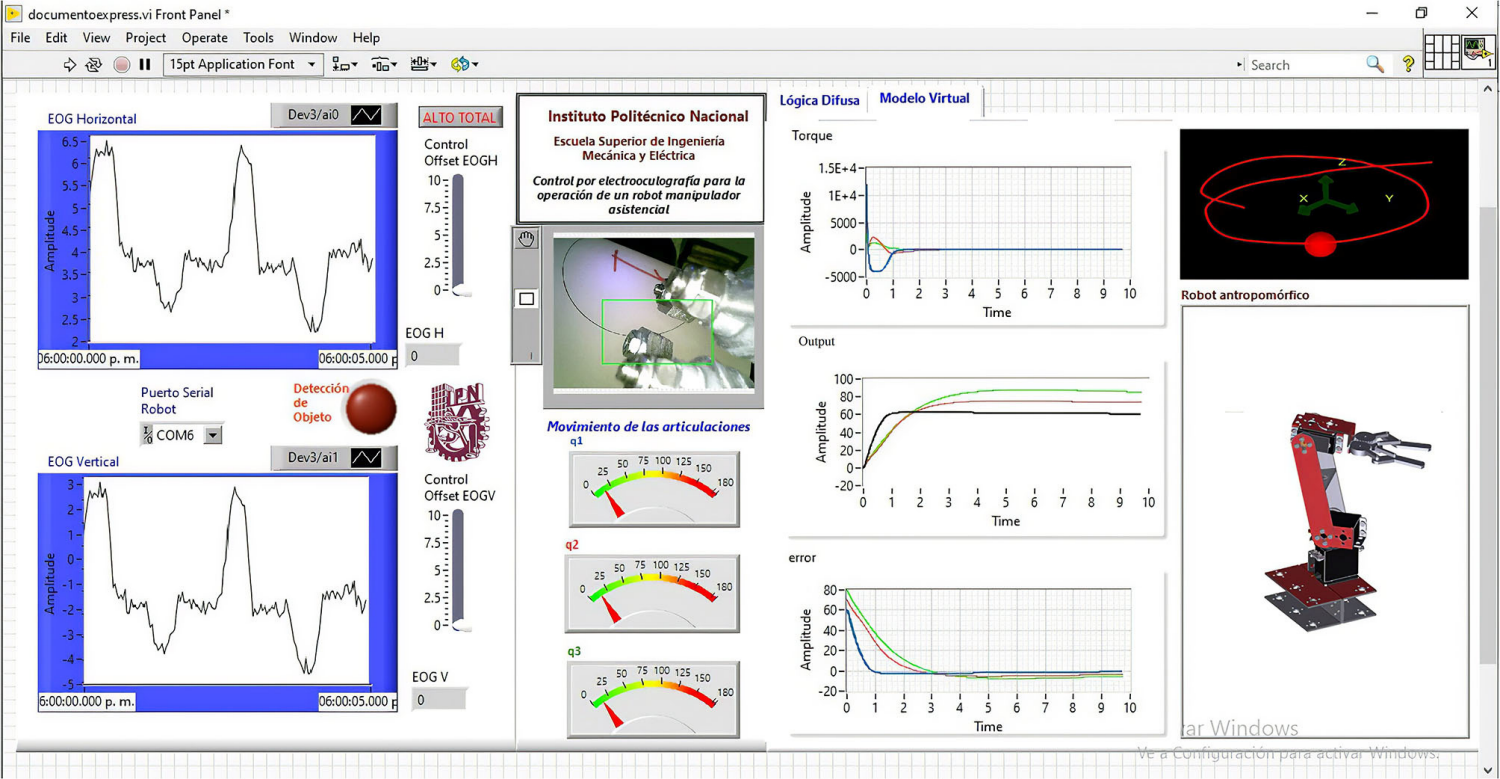
Graphical interface that provides system information to the user.

To perform different experiments to validate the operation of the designed HMI system, a graphical interface was developed that allows the operator to visualize the EOG signals of both channels, the movement of a virtual robot that emulates the movements generated by the interaction of the gaze, a graph showing the position in Cartesian coordinates of the data generated by the fuzzy classifier, and a visual feedback of the object to be taken by means of the image acquired by an external camera placed on the end effector. In addition, the response of the control algorithm, the position error, and the torque graph in each of the robot's joints are presented in the graphic interface (see Figure 12).

The characteristics of the robot used in the experiments are shown in Table 3.

**Table 3:** Physical characteristics of the robot.

**Physical robot dimensions**	**Position range of robot joints**	**Robot implemented**
*l*_1_ = 0.18 *m*	*q*_1_ = 20 − 70°	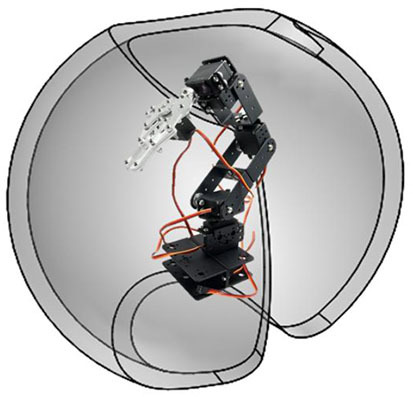
*l*_2_ = 0.28 *m*	*q*_2_ = 30 − 230°	
*l*_3_ = 0. 15 *m*	*q*_3_ = 20 − 270°	

To evaluate the performance of the HMI system, experiments were conducted with 60 individuals inexperienced in the use of this type of system. The purpose was to demonstrate that a system that adapts to the user allows a learning curve that requires fewer repetitions and therefore less time to perform a defined task, with the advantage of reducing the training time of a user to become an expert.

**Figure 13:**
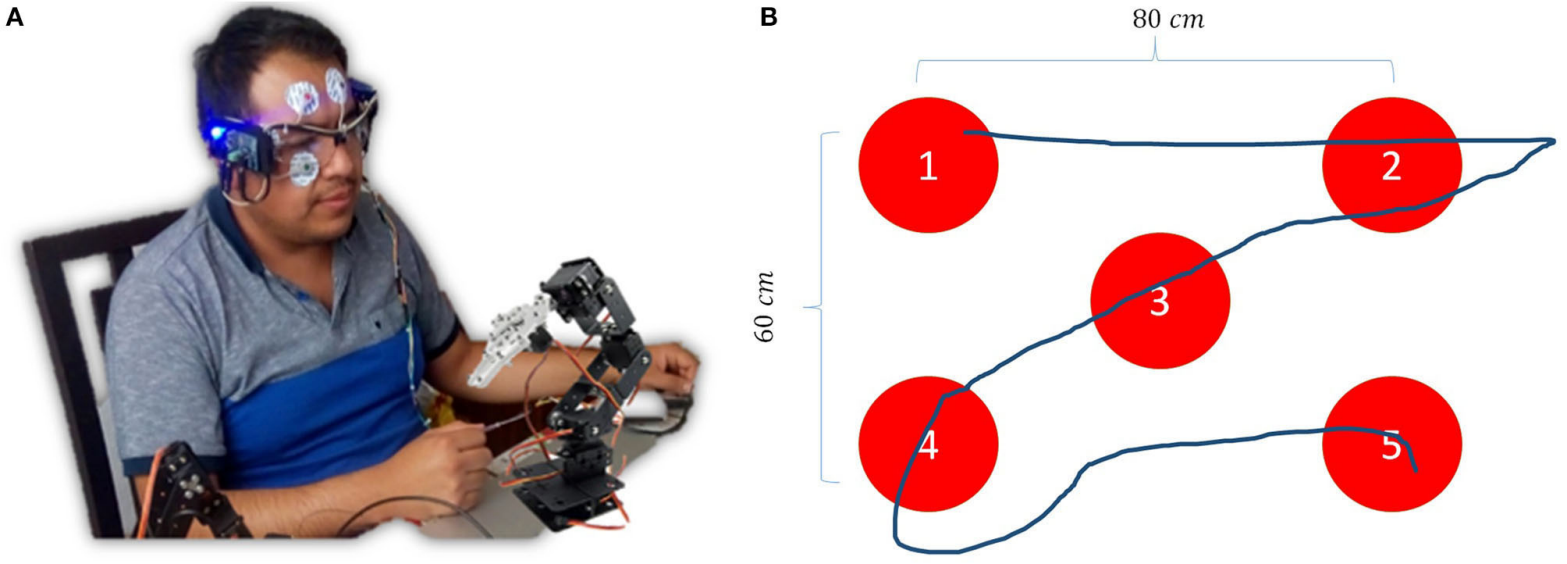
**(A)** Control of the physical manipulator robot by EOG. **(B)** Proposed trajectory for the validation of the system by following the gaze.

The performance of the HMI is verified by obtaining the time it takes the user, using the orientation of his eyeball (see Figure 13A), to control the robot to follow a trajectory defined by 5 points (Figure 13B). Each user performs twenty repetitions. A camera is placed on the end effector, and a program for detecting red color is added to the interface in real time. Each point has an internal number that defines the order that the robot must follow to indicate them; when the first red color point is detected, a timer is activated to take the time of the execution of the task. For evaluating adaptability of the classifier, it was necessary to compare the time of the execution of the twenty repetitions of the 30 users, in each experiment.

In the first experiment, the glasses are placed on each user and a sample of the EOG signal is taken for 25 s to generate a database with the 30 users, and the average voltage of the EOG signals is calculated for the maximum and minimum threshold values. The system is calibrated once, and all users need to do a workout to reach the required thresholds. In other words, in this first experiment, the user must adapt to the HMI in order to operate the assistance system.

In the second experiment, the system, by optimizing the signal thresholds, is automatically calibrated every 3 min, adapting the fuzzy classifier to the parameters of the EOG signal of each individual. The optimal range data becomes the input of the classifier; the calibration process is imperceptible to the user and does not affect the operation control of the assistance system since it only lasts 0.53 s. In this second experiment, the HMI adapts to each user and the variability of its EOG signal.

A third experiment was realized with the users of the second experiment, who had previous training to analyze the performance of users with experience in executing the task and evaluate if with only 20 training tests the execution time of this is considerably reduced.

### Experiment 1: Standard Calibration With Inexpert and Expert Users

In this experiment, 30 different EOG signals were obtained. The average of the maximum and minimum thresholds of the user voltage was calculated; the result gave a value of 1.123 volts for the maximum threshold and 0.3212 volts for the minimum threshold. The fuzzy classifier was calibrated with these databases, and the same 30 users were asked to perform the test. When making the first attempts, the users were unable to control the operation of the robot and complete the trajectory; it was necessary to do prior training in the use of the HMI and to manually adjust the thresholds of the fuzzy classifier for each user on the average value obtained for get them to complete the test. When they had the necessary training, time was taken in 20 repetitive tests.

**Figure 14:**
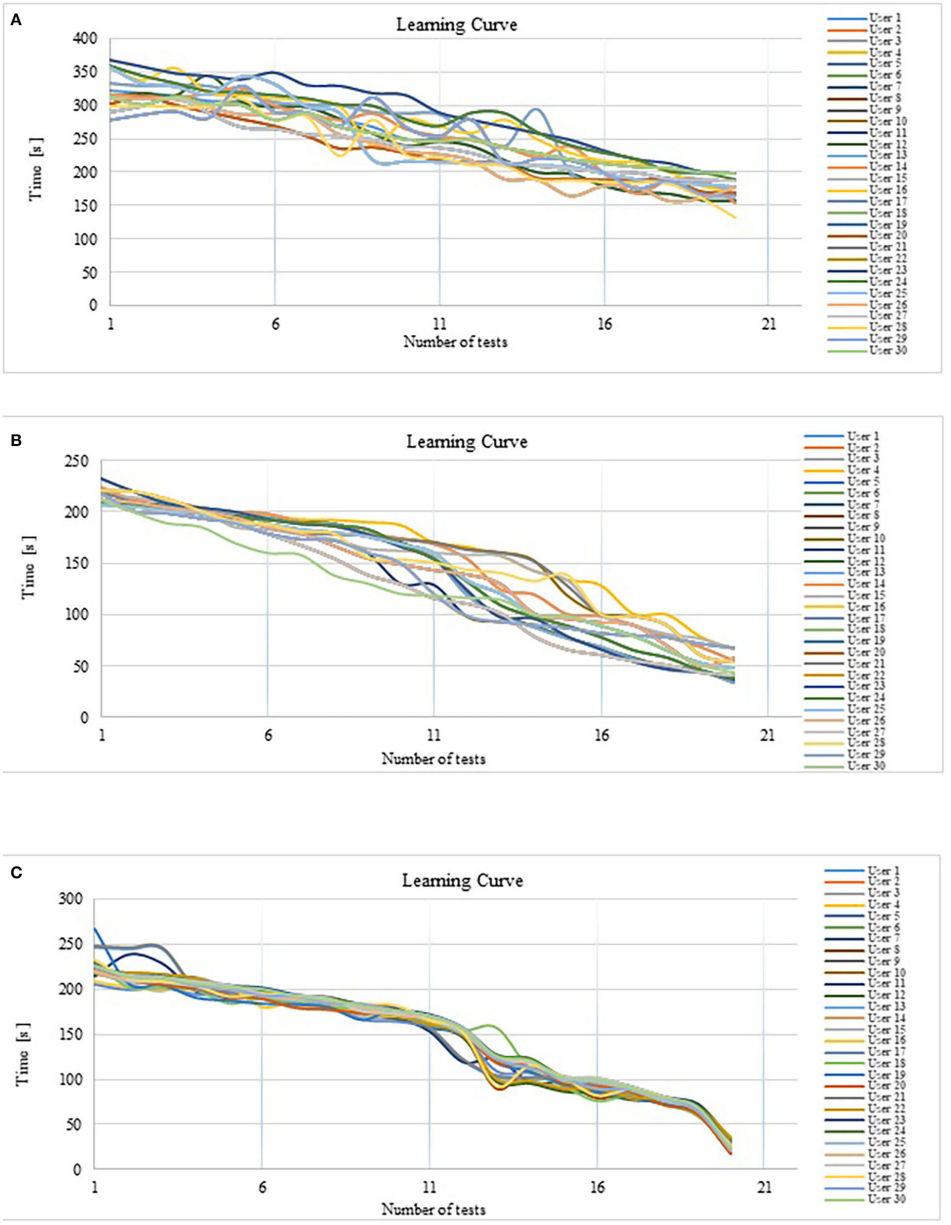
**(A)** Response time trend without optimization of the fuzzy classifier. **(B)** Response time trend with fuzzy classifier optimization. **(C)** Response time trend with fuzzy classifier optimization and expert users.

As seen in Figure 14A when starting the experiment, the average execution time of the task was 322.22 s; after 20 repetitions, the average was 175.7 s. In Figure 14A, the tendency to decrease the execution time to realize the path tracking is observed. Task execution time average was reduced by 45.5% after twenty tests. The standard deviation of the recorded time is 55.56, which indicates that there is considerable variation in relation to the average. This is because each user tries to adapt to the thresholds already preestablished in the system.

Table 4 shows the response time of a sample of 7 users out of 30 users who carried out the experiment. The time it takes to perform the 5-point tracking experiment is represented in the rows for each user. While in the column, the number z test is indicated.

**Table 4:** Value of the response trend of inexperienced users by manual calibration of the fuzzy classifier.

	**Response in time of experimental tests**																			
	**Test 1**	**Test 2**	**Test 3**	**Test 4**	**Test 5**	**Test 6**	**Test 7**	**Test 8**	**Test 9**	**Test 10**	**Test 11**	**Test 12**	**Test 13**	**Test 14**	**Test 15**	**Test 16**	**Test 17**	**Test 18**	**Test 19**	**Test 20**
User 1	322.90	319.20	315.32	307.38	306.38	300.12	298.13	280.34	268.38	250.34	249.28	239.83	218.39	200.38	198.38	180.38	170.39	168.38	158.82	158.39
User 5	358.38	343.38	332.39	320.29	318.38	314.38	309.39	300.28	298.39	278.38	268.39	288.29	287.38	258.39	239.29	228.38	218.39	200.39	198.39	188.39
User 10	278.39	286.45	291.38	281.39	329.69	290.89	289.98	270.14	312.45	267.89	254.32	278.98	214.38	220.86	218.43	200.78	176.45	187.89	167.56	165.90
User 15	368.38	358.38	348.28	344.38	339.30	349.38	330.39	329.38	318.38	315.38	289.38	278.38	268.38	258.38	248.38	232.38	219.28	213.29	200.29	198.28
User 20	300.89	312.33	299.80	289.08	278.65	268.54	253.40	234.57	236.78	228.12	217.45	214.37	212.38	190.29	189.76	187.45	188.09	189.45	170.45	169.02
User 25	357.38	332.90	328.38	316.38	343.38	332.29	298.38	288.39	218.29	216.39	215.39	214.59	213.38	210.12	208.32	198.34	189.54	184.38	182.18	178.38
User 30	312.76	300.45	312.40	300.75	299.89	278.13	288.12	267.34	258.54	248.80	246.50	248.90	236.78	228.76	219.48	213.45	208.54	206.43	200.12	198.34

In the analysis of the results, it is observed that there is a decrease in the response time resulting after test number 6, but a dispersion in the trend is observed, that is, the user did not achieve a good control of the operation of the robot until the repetition number 16. In test 16, it is observed how this dispersion decreases. This variability explains why the system does not respond adequately until the user reaches the voltage thresholds at which the classifier is calibrated. Most HMIs work on this principle; they are calibrated using information stored in a database, even if the user has different parameters from those stored, the system responds with close values, increasing the time in which a control command is generated because a search must be made for the closest parameter and then generate a response by activating the actuators of the system to be controlled. In addition, extensive user training is required to adapt as quickly as possible to the HMI calibration parameters.

### Experiment 2: Customized Calibration With Inexpert Users

For the second experiment, the times it took to perform the test for 30 new users were obtained, but in this case, the intelligent calibration system developed from the modeling of the voltage thresholds was used. The fuzzy inference system is automatically calibrated for each user every 3 min, from the first EOG signal acquisition until the test ends. This is a parallel process, and interrupting the control routine for a period of 0.53 s, the genetic algorithm obtains the thresholds and the optimal range is the new fuzzy classifier input, customizing the system and adapting the control to individual parameters, including when there are disturbances in the EOG signal due to external interference. The interruption time for calibration is imperceptible by the user and negligible, compared to the response time of the controlled device. The user does not require prior training to generate some skill in controlling the device, because the classifier is constantly calibrating.

In this experiment, the average time it took the 30 users to follow the path when starting the test was 215.53 s; after a series of 20 repetitions, the average execution time was 48.51 s. In Table 5, a summary of the response time of Experiment 2 is presented and it can be observed that the execution time is much less than the average obtained in the first experiment. As seen in Figure 14B, from the first test there was a tendency to decrease the average time by 77.55%, a value considerably higher than that observed in the first experiment. The standard deviation of the recorded time is 41.3, which indicates less variability in the response of different users using the optimal calibration for the classifier. In Figure 14B, can be see that the standard deviation is reduced after of test number 18, this indicates that the dispersion of the data is decreased, which suggests that all the users adapted to the system at the end of the Experiment 3: Customized calibration with expert users.

**Table 5:** Response trend value of inexperienced users through automated calibration of the fuzzy classifier.

	**Response in time of experimental tests**																			
	**Test 1**	**Test 2**	**Test 3**	**Test 4**	**Test 5**	**Test 6**	**Test 7**	**Test 8**	**Test 9**	**Test 10**	**Test 11**	**Test 12**	**Test 13**	**Test 14**	**Test 15**	**Test 16**	**Test 17**	**Test 18**	**Test 19**	**Test 20**
User 1	210.23	208.27	206.32	200.23	196.34	194.76	190.56	188.90	178.56	170.67	155.23	120.23	100.23	89.34	78.34	68.57	58.34	49.47	46.45	34.33
User 5	232.87	219.89	208.45	204.33	200.56	193.45	188.49	186.43	178.45	166.45	154.32	123.38	97.33	96.34	78.66	66.34	54.33	46.72	44.22	36.40
User 10	232.87	219.89	208.45	204.33	200.56	193.45	188.49	186.43	178.45	166.45	154.32	123.38	97.33	96.34	78.66	66.34	54.33	46.72	44.22	36.40
User 15	217.38	214.34	210.32	202.34	185.54	183.34	178.02	173.43	164.33	162.34	160.34	158.22	157.39	142.57	133.67	99.59	89.32	80.78	76.32	66.12
User 20	212.57	200.45	199.37	193.45	189.45	178.45	167.34	154.45	138.45	129.34	115.45	110.39	100.98	78.49	65.45	60.45	54.45	50.56	43.23	40.45
User 25	207.33	205.43	201.33	198.47	189.32	188.39	183.22	180.42	176.59	168.93	160.46	133.58	120.78	100.47	98.49	89.43	79.78	65.43	53.39	48.34
User 30	213.34	200.23	189.34	185.89	170.34	160.34	158.48	138.34	130.45	120.23	118.45	116.23	114.43	100.09	98.34	89.34	78.34	65.43	50.54	43.23

A third experiment was carried out with the 30 users who carried out the second experiment, and very significant results were obtained. In Table 6, a summary of the response time of Experiment 3 is presented. The average response time when starting the test of the 30 users is 224.15 s, after a series of 20 repetitions, it is verified that the tendency to decrease the execution time has an average of 24.09 s. With previous training, the average time to follow a new path decreased the response time of the robot by 89.26%, it can be observed in the graphs in Figure 14C. When analyzing the results presented, a significant improvement in the dispersion of the response is observed due to the decrease in the standard deviation of 29.8, which indicates a greater domain in the control of the system by users, especially after test 16. In Figure 14C, in the last two tests in the 30 users, a significant decrease in the execution time of the task is observed.

**Table 6:** Response trend value of expert users by automated calibration of the fuzzy classifier.

	**Response in time of experimental tests**																			
	**Test 1**	**Test 2**	**Test 3**	**Test 4**	**Test 5**	**Test 6**	**Test 7**	**Test 8**	**Test 9**	**Test 10**	**Test 11**	**Test 12**	**Test 13**	**Test 14**	**Test 15**	**Test 16**	**Test 17**	**Test 18**	**Test 19**	**Test 20**
User 1	245.49	243.48	243.93	200.23	196.34	194.76	190.56	188.9	178.56	170.67	155.23	120.23	102.23	99.99	100.34	97.56	86.46	76.12	66.59	30.19
User 5	220.19	209.38	208.45	204.33	200.56	193.45	188.49	186.43	178.45	172.03	166.28	151.23	122.98	119.28	98.38	97.32	88.28	76.38	65.38	21.99
User 10	224.35	212.24	210.78	204.57	200.35	198.45	190.78	186.78	179.49	174.45	168.89	154.34	124.45	119.56	100.34	98.56	89.46	78.34	68.43	23.38
User 15	219.19	206.38	197.45	212.33	200.56	189.45	178.49	176.43	169.45	170.03	164.28	150.23	119.98	117.28	93.38	94.32	86.28	72.38	63.38	17.99
User 20	221.35	210.24	204.78	200.57	193.35	189.45	179.78	177.78	173.49	172.45	168.89	151.34	119.45	116.56	96.34	93.56	86.46	72.34	62.43	18.38
User 25	220.19	209.38	208.45	204.33	200.56	193.45	188.49	186.43	178.45	172.03	166.28	151.23	122.98	119.28	98.38	97.32	88.28	76.38	65.38	21.99
User 30	225.52	213.41	211.95	205.74	201.52	199.62	191.95	187.95	180.66	175.62	170.06	155.51	125.62	120.73	101.51	99.73	90.63	79.51	69.6	24.55

With this HMI, the user does not have to worry about reaching the required voltage levels or need prior training to control the robot, on the contrary, the HMI adapts to the operating thresholds of each user, generating a response from the robot throughout its workspace.

## Conclusion

In this work, an intelligent calibration system is presented by means of which an HMI interface whose control input is the EOG signal adapts to the characteristics of the signals of different users and generates trajectories in the workspace of an anthropomorphic robot manipulator in real time. The difference from other HMIs is that the proposed system does not need a database for its calibration. The innovation is the intelligent system capable of calibrating the HMI from the use of fast neural networks to model the physiological signal and its optimization with genetic algorithms to obtain amplitude thresholds that allow easy adaptation of the HMI to the EOG signal of the user. It is verified that the use of artificial intelligence to generate trajectories from signals with high variability, such as EOG results in a decrease in the execution time of a task and the sensation of real-time control of the robot. It was shown, from the observation of data obtained by experimentation, that the adaptive calibration system generates response times in the robotic system to be controlled less than when the user is trained to use standard calibrated systems. When comparing Figure 14C with Figure 14A, the decrease in task execution time is observed. For the first experiment (users with manual calibration experience), the average decrease in task execution time is 44%, and for the third experiment (users with adaptive calibration experience) it is 82%. In addition, using an intelligent system reduces training time, since the user does not have to adapt to the HMI if not the HMI adapting to the user. In Figure 14A, a large dispersion of data is observed (standard deviation), indicating that each user tries to adapt differently to the HMI. In contrast, Figure 14C shows a significant reduction in data dispersion (standard deviation), since each user manages to control the system adequately, since the HMI adapts to the characteristics of its EOG signal.

In the graphs of Figure 14, it is observed how the adaptability of the system improves; in the first experiment, the calibration was done manually, although it presents a decrease in response time, where it takes the user more time to reach the objective set; however, the fuzzy logic allows adaptability to personal characteristics. The second experiment has been worked with inexperienced users who had no control over the system, but calibrating the system from modeling the signal and optimizing the range of signal variability, it is observed that the response time is less and the level of adaptability is verified by decreasing the measure of dispersion of each of the responses. The system tends to standardize the learning curve to the same pattern regardless of the individual who uses the HMI; this property of the modeling of the EOG signal to customizing the fuzzy classifier can be seen in the results of Experiment 3, in the graph of Figure 14C where the response time decreases to an average value of 24 s and the standard deviation measure is reduced.

The experiment was performed using an anthropomorphic robot to validate the HMI response, but since the fuzzy classifier generates coordinates in a Cartesian space (in three dimensions), it can be adapted to any navigation system by modifying only the mapping in the workspace, generating trajectories for example for autonomous vehicles or intelligent spatial location systems for the control of wheelchairs or any type of mobile robot.

In a future work, this HMI would be implemented in assistance systems for people with severe disabilities, by implementing an eye joystick system in order to accomplish everyday tasks, such as taking objects.

## Data Availability Statement

All datasets generated for this study are included in the article/supplementary material.

## Ethics Statement

Ethical review and approval was not required for the study on human participants in accordance with the local legislation and institutional requirements. The patients/participants provided their written informed consent to participate in this study. Written informed consent was obtained from the individual(s) for the publication of any potentially identifiable images or data included in this article.

## Author Contributions

FP, PN, OA, MC, EV, and EP conceived, designed, performed the experiments, analyzed the data, and wrote the paper. All authors contributed to the article and approved the submitted version.

## Conflict of Interest

The authors declare that the research was conducted in the absence of any commercial or financial relationships that could be construed as a potential conflict of interest. The reviewer LS declared a shared affiliation, though no other collaboration, with several of the authors FP, PN, MC, EV, and EP to the handling Editor.
